# Alterations in Skeletal Muscle mRNA Abundance in Response to Ethyl-Cellulose Rumen-Protected Methionine during the Periparturient Period in Dairy Cows

**DOI:** 10.3390/ani12131641

**Published:** 2022-06-26

**Authors:** Lam Phuoc Thanh, Qianming Jiang, Nithat Wichasit, Fernanda Batistel, Claudia Parys, Jessie Guyader, Juan J. Loor

**Affiliations:** 1 Department of Animal Sciences, Can Tho University, Ninh Kieu, Can Tho 94000, Vietnam; phuocthanh@ctu.edu.vn; 2 Department of Animal Sciences and Division of Nutritional Sciences, University of Illinois, Urbana, IL 61801, USA; qj5@illinois.edu (Q.J.); wichasit.n@gmail.com (N.W.); 3 Department of Agricultural Science, Naresuan University, Phitsanulok 65000, Thailand; 4 Department of Animal Sciences, University of Florida, Gainesville, FL 32608, USA; fernandabatistel@ufl.edu; 5 Evonik Operations GmbH, Hanau-Wolfgang, 63457 Essen, Germany; claudia.parys@evonik.com (C.P.); jessie.guyader@evonik.com (J.G.)

**Keywords:** dairy cows, mRNA abundance, periparturient, rumen-protected methionine, skeletal muscle

## Abstract

**Simple Summary:**

Improvements in performance around parturition by feeding ethyl-cellulose rumen-protected methionine (RPM) have been reported. Hepatic and adipose gene transcription effects have been a key focus of studies dealing with RPM. It is unknown, however, if RPM alters the abundance of genes associated with nutrient metabolism and biochemical pathways in skeletal muscle. This tissue also experiences dynamic biological changes around parturition. The objective was to use skeletal muscle biopsies from cows fed a control or RPM-supplemented diet around parturition to investigate alterations in mRNA abundance. Compared with controls, feeding RPM led to overall upregulation of genes associated with amino acid transport, carnitine transport, β-oxidation, vitamin transport, mTOR pathway, insulin signaling, antioxidant response, CDP-Choline pathway, and arginine metabolism. Thus, besides positive effects at the level of performance, RPM can help skeletal muscle maintain homeostasis around parturition partly through alterations in mRNA abundance.

**Abstract:**

This study aimed to evaluate the effect of feeding ethyl cellulose rumen-protected methionine (RPM) on skeletal muscle mRNA abundance during the periparturient period. Sixty multiparous Holstein cows were used in a block design and assigned to either a control or RPM diet. The RPM was supplied from −28 to 60 days in milk (DIM) at a rate of 0.09% (prepartum) or 0.10% (postpartum) of dry matter (DM), ensuring a Lys:Met in the metabolizable protein of ~2.8:1. Muscle biopsies were collected at −21, 1, and 21 DIM. Thirty-five target genes associated with nutrient metabolism and biochemical pathways were measured via RT-qPCR. The mRNA abundance of genes associated with amino acid (AA) transport (*SLC7A8*, *SLC43A2*), carnitine transport (*SLC22A5*), insulin signaling (*IRS1*), and antioxidant response (*NFE2L2*) had diet × time effect (*p* < 0.05) due to greater abundance in RPM versus CON cows, especially at 1 and 21 DIM. Members of the AA transport (*SLC7A8*, *SLC25A29*, *SCL38A9*), fatty acid β-oxidation (*ACADVL*), vitamin transport (*SLC5A6*, *SLC19A2*), mTOR pathway (*AKT1* and *mTOR*), antioxidant response (*KEAP1*, *CUL3*), CDP-Choline pathway and arginine metabolism had overall greater abundance (*p* < 0.05) in RPM versus CON cows. Overall, data indicate that RPM can alter nutrient metabolism in the skeletal muscle around parturition partly through alterations in mRNA abundance.

## 1. Introduction

Dairy cows undergo drastic physiological changes to support the rapidly growing fetus and lactation during the periparturient (“transition”) period. Post-parturition, cows enter a period of negative energy and protein balance because they cannot consume enough feed to support the requirements for milk synthesis [1]. To support the offspring (fetus or lactation), the cow may mobilize up to 80 kg of empty body (EB) lipid and 17 kg of EB protein (~40 and 27% of total EB stores, respectively) within the first 2 months after parturition [2,3]. Excessive protein or lipid mobilization may negatively influence cow health and milk production [4,5]. Even though extensive research focus has been placed on reducing negative energy balance, less research has been conducted on the consequences of indispensable amino acid (AA) imbalances. Published data has highlighted that lactation performance was improved by feeding ethyl-cellulose rumen-protected methionine (RPM) [6], rumen-protected lysine [7], or AA balanced-diets [8] around parturition.

The skeletal muscle, the largest organ in mammals, plays a major role in maintaining metabolic homeostasis and adaptations to the physiological needs of pregnancy and lactation [9]. However, unlike the liver and adipose tissue, the metabolic and regulatory role of skeletal muscle in the adaptation of dairy cows to early lactation has not been studied extensively. A recent report demonstrated that the abundance of skeletal muscle genes related to protein synthesis, energy and AA metabolism, inflammation and oxidative stress, and muscle fiber type was altered when Holstein cows were fed individual rumen-protected AA from −14 d to 25 d relative to calving [10]. Methionine typically is the first-limiting AA for lactating cows [1], with studies reporting beneficial effects of RPM on performance and immunometabolic status during the transition period [6,11]. Although the effects of RPM feeding prior to parturition on mRNA abundance in the liver have been reported [12], to our knowledge, it is unknown if feeding RPM alters the abundance of genes associated with nutrient metabolism and biochemical pathways in skeletal muscle.

The growing body of research in the area of AA nutrition of transition cows underscores the need for a greater understanding of the potential effects of these nutrients at the cellular level in key organs of the cow. Thus, besides the historical focus on the liver in the context of feeding RPM, a key question is whether an enhanced post-ruminal supply of Met affects important molecular pathways in skeletal muscle. For instance, if RPM alters molecular mechanisms in the muscle that are associated with fundamental functions such as nutrient transport and metabolism or insulin responsiveness, such data would help fine-tune the nutrition of cows around parturition.

Our general hypothesis was that the abundance of genes associated with nutrient metabolism (transport of AA, vitamins, and fatty acids), biochemical pathways (*mTOR*/insulin signaling, CDP-Choline pathway, and arginine metabolism), and antioxidant responses would be increased alongside dry matter intake (DMI) when RPM was fed to cows [6]. The specific objective of this study was to use skeletal muscle biopsy tissue from cows fed ethyl-cellulose RPM in one of our previous studies [6] to investigate the mRNA abundance of genes during the periparturient period.

## 2. Materials and Methods

### 2.1. Animal Housing and Care

All experimental procedures were performed according to The Institutional Animal Care and Use Committee at the University of Illinois, USA (Urbana; protocol no. 14270). All cows were housed in a free-stall system equipped with a gate system (American Calan Inc., Northwood, NH, USA) during the prepartum period. They were then housed in tie-stalls during the post-partum period. Cows were fed once daily (13:00 h) at 120% of expected intake and milked three times daily (at 06:00, 14:00, and 22:00 h).

### 2.2. Experimental Design and Diets

A subset of cows with a complete set of biopsies from our published study were used [6]. These cows averaged 773 ± 97 kg body weight (BW) in the control and 785 ± 100 kg BW in the RPM group at 30 d from calving. Parity prior to calving averaged 2.86 ± 0.50 in controls and 3.05 ± 0.45 in RPM. Briefly, 60 multiparous Holstein Friesian cows from the University of Illinois Dairy Research Farm were arranged in a randomized complete block design experiment with 30 cows per treatment. Cows were blocked by the expected parturition day, and the blocks were balanced by parity, previous 305-d milk yield, and body condition score (BCS). Cows within each block were randomly assigned to 1 of the 2 treatments. Treatment diets were a basal control diet with no Met supplementation or the basal diet supplemented with ethyl-cellulose RPM (Mepron, Evonik Nutrition, and Care GmbH, Hanau-Wolfgang, Germany). Ethyl-cellulose RPM was supplied from −28 to 60 d relative to parturition at a rate of 0.09 and 0.10% DMI of the previous day during the prepartum and postpartum periods, respectively. These target values were based on previous experiments demonstrating a benefit in terms of production performance and health of supplementing RPM to achieve a Lys:Met ratio close to 2.8:1 during the prepartum and postpartum periods [13,14]. The intestinal digestibility coefficient and rumen bypass of RPM are 90% [15] and 80% [16]; therefore, the cows received 6.1 g of Met available for absorption/10 g of Mepron. Feed ingredients and chemical composition (dry matter, DM, basis) of the diets are reported in Table 1. Diets were mixed daily in a tumble mixer, and ethyl-cellulose RPM was top-dressed on the TMR. All rations were formulated to meet cow predicted requirements according to published recommendations [1].

### 2.3. Sampling

Skeletal muscle biopsies were harvested at −21, 1, and 21 d around parturition. Needle biopsy of semitendinosus muscle was performed in a subset of cows in the control and RPM groups, alternating sides of the thigh region after the morning milking. Briefly, the hair in a 20 × 20 cm^2^ area was shaved close to the skin prior to application of Dermachlor 4% surgical scrub (Butler Animal Health Supply, Dublin, OH, USA) solution for pre-surgical skin cleaning. Subsequently, a 5-mL lidocaine solution was injected subcutaneously and intramuscularly and a 1-cm incision made approximately 10 min after anesthesia. Approximately 500 mg of muscle tissue was collected using a needle biopsy instrument (BARD Magnum, 14 gauge × 16 cm; C. R. Brad, Inc., Murray Hill, NJ, USA). Muscle tissue was snap-frozen in liquid nitrogen until RNA extraction. A surgical staple was applied to close the incision and removed at 6 d post-biopsy as appropriate. A complete set of biopsies was obtained for 10 cows in the control and RPM groups and were used for transcript profiling.

### 2.4. RNA Extraction and Quality Assessment

The method used to extract RNA from muscle tissue was similar to standard procedures in our laboratory [17]. The extracted RNA was measured at concentration using a NanoDrop ND-1000 spectrometer instrument (Thermo Fisher, Waltham, MA, USA). Six mL of diluted RNA solution (100 ng/µL) was then used for RNA quality analysis in a Fragment Analyzer (Advanced Analytical Technologies Inc., Orangeburg, NY, USA). Samples for subsequent analysis had an RNA quality number (RQN) ≥7.0. One hundred ng of RNA was used for cDNA synthesis.

### 2.5. cDNA Synthesis

Total RNA was diluted to a concentration of 100 ng/μL. Master Mix 1 (MM1) and Master Mix 2 (MM2) solutions were prepared following standard protocols in our laboratory [17]. In a 200 µL tube, 10 µL of MM1 was mixed with 1 µL diluted RNA sample (100 ng/μL) and incubated at 65 °C for 5 min. Nine µL of MM2 was then added to the solution, and the tubes were incubated using as described previously [17]. The cDNA samples were then diluted (1:4 *v*:*v*) with molecular biology grade water (AccuGene; Cat No: 51200) and stored at −80 °C.

### 2.6. Primer Design and Testing

The cDNA sequence for each gene of interest was retrieved from the National Center for Biotechnology Information (NCBI, accessed on 15 November 2021; https://www.ncbi.nlm.nih.gov/). Gene sequences with amplicon sizes closest to 100 base pairs were chosen. The Basic Local Alignment Search Tool (BLAST) from NCBI for *Bos taurus* was used with default settings to test the designed primer sequences. Primers were manufactured by Integrated DNA Technologies (https://www.idtdna.com). Primer sequences developed in the presented study are listed in Table S1. Primers for *SLC38A6* [18], *SLC38A9* [19], *CPT1A*, *ACADVL* [20], *NFE2L2*, *KEAP1*, [21], *CHKA*, *CHKB*, *PCYT1A*, *PCYT1B*, *CEPT1* [22], *ODC1*, *SRM*, *AMD1*, *ARG1*, *SMS*, *NOS2* [23], and *NOS3* [24] were obtained from published papers.

Primers were tested following the method described previously in one of our manuscripts [25]. The purified PCR products were sequenced at the University of Illinois Core Sequencing facility. The sequencing results were searched against the *Bos Taurus* database using NCBI Nucleotide BLAST and default settings. The sequencing results that matched the primer’s sequence are reported in Tables S2 and S3.

### 2.7. Real-Time qPCR

Four µL negative controls, standard curve, and diluted cDNA samples were pipetted into their respective wells in a MicroAmp™ Optical 386-well reaction plate in duplicate. Six µL of SYBR Green Master Mix (VWR; Cat No 101414-276) was then pipetted in each well. The PCR reaction was performed in a QuantStudio 7 real-time PCR machine (Applied Biosystems, Waltham, MA, USA) under the following conditions: 2 min at 50 °C, 10 min at 95 °C, and 40 cycles of 15 s at 95 °C, followed by 1 min at 60 °C. The RT-qPCR data were then analyzed using the QuantStudio™ Real-Time PCR Software (version 1.7.1, Applied Biosystems, Waltham, MA, USA). Before statistical analysis, RT-qPCR data were normalized using the geometric mean of the internal control genes, including glyceraldehyde 3-phosphate dehydrogenase (*GAPDH*), ribosomal protein S9 (*RPS9*), and ubiquitously expressed prefoldin-like chaperone (*UXT*).

### 2.8. Statistical Analysis

The MIXED procedure of SAS version 9.4 (SAS Institute Inc., Cary, NC, USA) was used for repeated measures analysis of mRNA abundance. The fixed effects in the model were diet and time, and the random effect was cow. Compound symmetry (CS) was the most-appropriate covariate structure used for analysis. The degrees of freedom were corrected using the Kenward-Roger (KR) method, which yields more precise and efficient estimates of the fixed effects in experiments with a moderate to small sample size. Differences were considered statistically significant when *p* ≤ 0.05 and considered a trend when 0.05 < *p* ≤ 0.10.

## 3. Results and Discussion

### 3.1. Percentage mRNA Abundance

Table S4 depicts the RT-qPCR performance of all measured genes. Relative mRNA abundance, which is the individual proportion of mRNA present in skeletal muscle compared to the abundance of all measured genes, showed that *SLC38A2* (AA transporter) had the greatest relative mRNA abundance (16.6%), followed by *SLC43A2* (AA transporter) with 11.3%, and the lowest one was observed for *ARG1* (0.02%) and *NOS2* (0.03%). The relative mRNA abundance was quite high in genes associated with insulin signaling (IRS1, 5.09%), antioxidant response (*NFE2L2*, 4.15%; *KEAP1*, 6.87% and *CUL3*, 6.96%), fatty acid β-oxidation (*ACADVL*, 7.83%), CDP-Choline pathway (*CHKB*, 4.93%) and arginine metabolism (*ODC1*, 9.74%), but quite low in mTOR signaling (*AKT1*, 2.73%; *mTOR*, 1.41%) and vitamin-transporters (*SLC5A6*, *SLC19A2* and *SLC44A1*; 0.20–1.07%).

### 3.2. Amino Acid Transport

**Figure 1:**
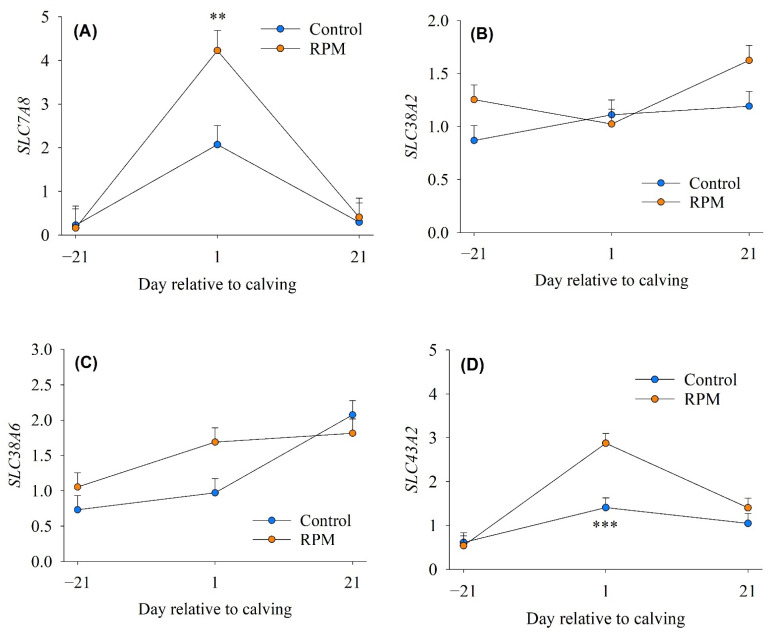
mRNA abundance of genes associated with amino acid transport in skeletal muscle of dairy cows (*n* = 10/diet) fed a basal control diet or the basal diet plus ethyl-cellulose rumen-protected methionine (RPM) from −21 to 21 day of calving. (**Panel A**), solute carrier family 7 member 8 (*SLC7A8*); (**Panel B**), solute carrier family 38 member 2 (*SLC38A2*); (**Panel C**), solute carrier family 38 member 6 (*SLC38A6*); (**Panel D**), solute carrier family 43 member 2 (*SLC43A2*). Error bars represent SEM. ** *p* < 0.01, *** *p* < 0.001.

There was a diet × time interaction for *SLC7A8* (*p* = 0.035) and *SLC43A2* (*p* = 0.006) (Table 2). At the 1st day of calving, the abundance of *SLC7A8* and *SLC43A2* was greater (*p* = 0.001 and *p* < 0.0001) in RPM (4.23 and 2.88) compared with controls (2.07 and 1.41) (Figure 1A,D). The overall abundance of *SLC25A29* and *SCL38A9* with RPM was 1.98- and 1.44-fold greater compared with the control (Table 2). In cows fed typical dry period diets without RPM, concentrations of plasma Met and the nonessential amino acids (NEAA) Asp, Gln, Glu, Cys, Tyr, and Orn as well as glucose decrease continuously from −25 d relative to calving and remain lower in early lactation [26]. Furthermore, when compared to the 3rd week pre-partum, in the 4th week post-partum, skeletal muscle concentrations of essential amino acids (EAA), Gln/Glu, Asp/Asn, Tyr, and Ser were decreased to ∼86% [26]; thus, besides helping cows achieve optimal rates of DMI [6,14,27,28], supplementing Met, first-limiting AA for dairy cows, during the transition period could help prevent drastic decreases in muscle Met. Biologically, the supply of Met in skeletal muscle could play a key role in the initiation of translation, i.e., synthesis of Met-tRNA for binding to the initiation codon AUG [29] especially during a time when the cow still is experiencing a state of negative nutrient balance.

The movement of AA across cellular membranes occurs mainly through active transport processes, and these proteins are generally classified as accumulative transporters and exchangers [30]. Accumulative AA transporters enhance AA concentrations by driving influx against their concentration gradient, commonly by co-transporting extracellular sodium, whereas exchangers alter the intracellular AA composition by swapping between intra- and extracellular compartments [30]. The greater abundance of *SLC7A8* in response to feeding RPM and increasing Met supply suggested a greater ability for uptake of branched-chain AA (Ile, Leu, and Val) and aromatic AA (Tyr and Phe); whereas the greater mRNA abundance of *SLC43A2*, *SLC25A29*, and *SCL38A9*, which are involved in the transport of some essential and non-essential AA, indicated a greater ability for uptake of Met, Lys, Leu, Phe, Val (all essential AA), and Arg (non-essential AA) in cows fed RPM. A previous study reported greater mRNA abundance of branched-chain and aromatic AA transporters (*SLC7A5*) and Leu, Phe, Val, and Met transporters (*SLC43A2*) in term placentomes when dairy cows were fed RPM during late-gestation [18]. Taken together, these data suggested that feeding RPM and the positive effect on DMI led to the upregulation of genes associated with the transport of both essential AA and non-essential AA. The degradation of muscle protein contributes to the accumulation of free AA in muscle cells during early lactation, and increased DMI after the second week of lactation helps support a return to basal concentrations of most of the free AA that decreased around parturition [26]. As such, the present data led us to speculate that skeletal muscle remodeling might have been enhanced in response to feeding RPM.

### 3.3. Carnitine Transport and β-Oxidation

**Figure 2:**
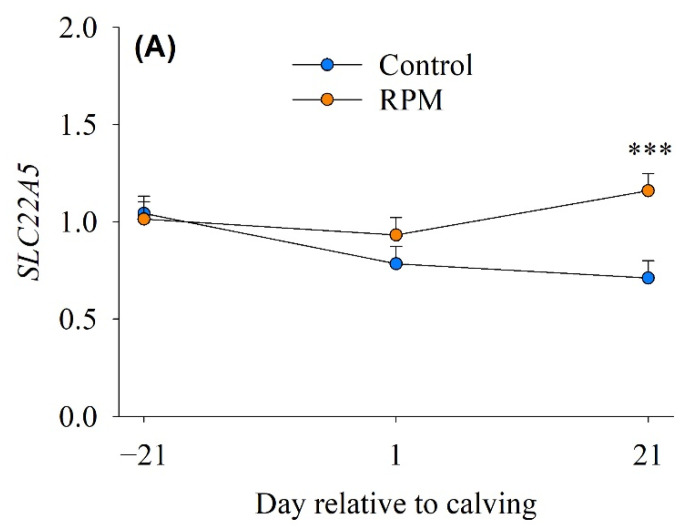

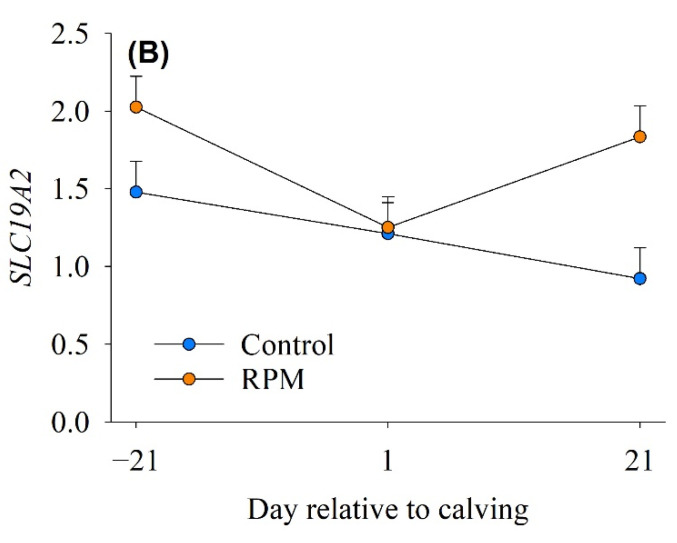
mRNA abundance of genes associated with carnitine and vitamin transport in skeletal muscle of dairy cows (*n* = 10/diet) fed a basal control diet or the basal diet plus ethyl-cellulose rumen-protected methionine (RPM) from -21 to 21 day of calving. (**Panel A**), solute carrier family 22 member 5 (*SLC22A5*); (**Panel B**), solute carrier family 19 member 2 (*SLC19A2*). Error bars represent SEM. *** *p* < 0.001.

There was diet × time interaction for *SLC22A5*, (*p* = 0.022; Table 2), a carnitine transporter. Compared with controls (0.71), cows fed RPM had greater (*p* = 0.001) abundance of *SLC22A5* (1.16) at 21 d post-partum (Figure 2A). In skeletal muscle, carnitine plays an essential role in the translocation of long-chain fatty acids (LCFA) into the mitochondrial matrix for subsequent β-oxidation and in the regulation of the mitochondrial acetyl-CoA/CoASH ratio [31]. Work with mice demonstrated that the protein encoded by *SLC22A5* is the main carnitine transporter in the small intestine [32]. Thus, the greater mRNA abundance of *SLC22A5*, which specifically transports carnitine, in response to feeding RPM indicated that skeletal muscle in these cows had a greater capacity for LCFA transport from the cytosol into the mitochondria for subsequent oxidation to generate ATP [33]. As such, this tissue might have been less reliant on glucose for energy, hence, allowing for its preferential use by the mammary gland for milk synthesis.

There was a positive effect (*p* = 0.068) of RPM on mRNA abundance of *CPT1A*, the rate-limiting enzyme in FA β-oxidation; further, RPM led to greater (*p* = 0.013) mRNA abundance of *ACADVL* compared with controls (1.36 vs. 0.98; Table 2). Mobilization of adipose tissue began before parturition, causing increased plasma LCFA and decreased plasma glucose concentrations [4]. Increased oxidation of FA for ATP production in skeletal muscle could be a mechanism to spare glucose for the fetus and colostrogenesis. Compared with 3 and 30 d post-partum, the abundance of *CPT1A* in skeletal muscle of dairy cows was greater at −17 d relative to parturition [34]. A similar effect was reported [10] in skeletal muscle *CPT1A* at −15 d prepartum compared with 0 d relative to parturition. Clearly, in the present study, increased *CPT1A* and *ACADVL* after calving suggested that the capacity to transport LCFA into the mitochondria for β-oxidation was upregulated by feeding ethyl-cellulose RPM.

### 3.4. Vitamin Transport

There was no significant diet × time interaction for vitamin-transporters, including *SLC5A6*, *SLC19A2*, and *SLC44A1* (Table 2). However, mRNA abundance of *SLC5A6* and *SLC19A2* was greater (*p* = 0.006 and *p* = 0.013) with RPM (1.86 and 1.70) relative to controls (1.14 and 1.20). Expression of *SLC44A1* tended to be greater (*p* = 0.082) with RPM compared with controls (1.25 vs. 1.05). The mRNA abundance of *SLC5A6* in the placentome tended to be greater in dairy cows fed ethyl-cellulose RPM during late gestation [18]. Thus, the greater mRNA abundance of genes associated with multivitamin transporter might have allowed more efficient uptake of these nutrients by skeletal muscle.

### 3.5. mTOR/Insulin Signaling

**Figure 3:**
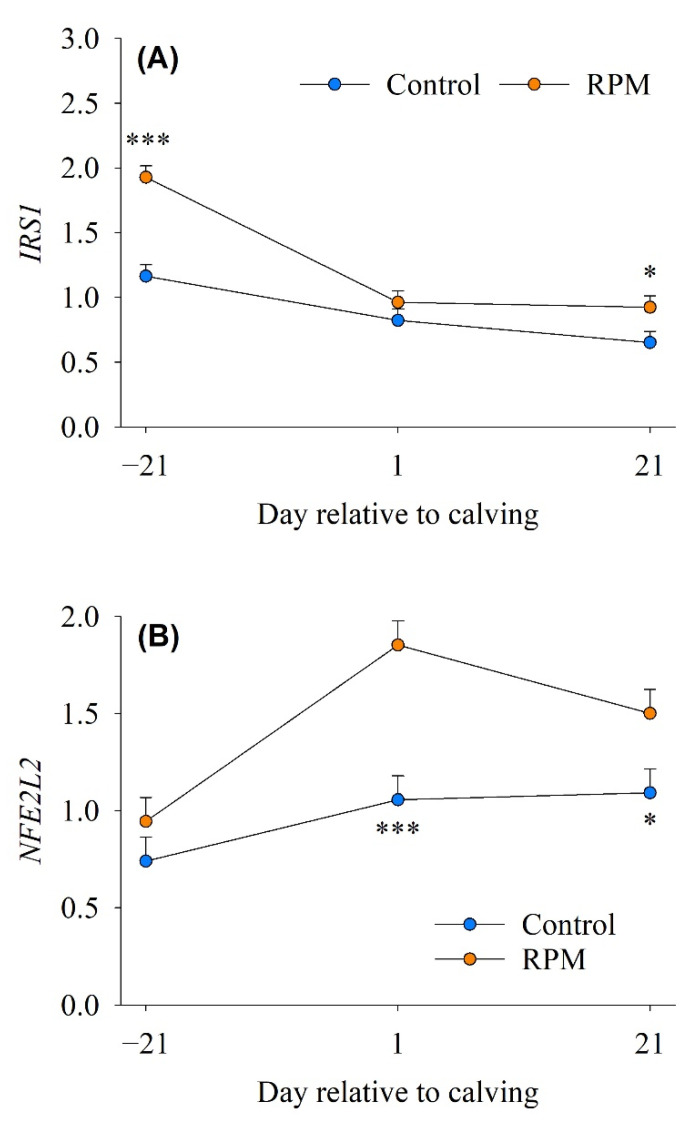
mRNA abundance of genes associated with insulin signaling and antioxidant response in skeletal muscle of dairy cows (*n* = 10/diet) fed a basal control diet or the basal diet plus ethyl-cellulose rumen-protected methionine (RPM) from −21 to 21 day of calving. (**Panel A**), insulin receptor substrate 1 (*IRS1*); (**Panel B**), NFE2 like bZIP transcription factor 2 (*NFE2L2*). Error bars represent SEM. * *p* < 0.05, *** *p* < 0.001.

No diet × time interaction was detected for genes involved in *mTOR* signaling (*AKT1* and *mTOR*), but RPM led to greater mRNA abundance of *AKT1* (*p* = 0.006) and *mTOR* (*p* = 0.033) compared with controls (Table 2). There was a diet × time interaction (*p* = 0.002) for insulin receptor substrate 1 (*IRS1*). The abundance of *IRS1* with RPM was greater than controls at −21 d (1.93 vs. 1.16; *p* < 0.0001) and 21 d (0.92 vs. 0.65; *p* = 0.033) relative to calving (Figure 3A). In nonruminants, it is well known that the *mTOR* signaling pathway is a key regulator of protein synthesis, cell growth, and proliferation [35]. The *mTOR* is a conserved serine/threonine kinase located downstream of phosphoinositide 3-kinase, while *AKT1* regulates cell development and proliferation via activation of the ribosomal protein S6 kinase to phosphorylate ribosomal protein S6, which consequently regulates protein synthesis, among other functions [36,37]. Thus, greater *AKT1* and *mTOR* suggested that cell growth and protein synthesis might have been enhanced by feeding RPM. Glucose and individual AA such as Leu and Arg can regulate *mTOR* signaling independently [38]. A greater post-ruminal supply of Met was associated with greater plasma insulin in cows [18]; thus, we speculate that the availability of Met and essential AA might stimulate the mTOR signaling pathway, leading to greater AA transport into skeletal muscle. In the present study, the upregulation of IRS1 due to feeding RPM would indicate greater insulin sensitivity [39], an idea supported by the greater rate of DMI in those cows [6].

### 3.6. Antioxidant Response

A diet × time interaction (*p* = 0.010) was detected for genes associated with transcriptional control of antioxidant responses (*NFE2L2*) (Table 2). Compared with control, cows fed RPM had greater abundance of *NFE2L2* at 1 d (1.85 vs. 1.06; *p* < 0.0001) and 21 d after calving (1.50 vs. 1.09; *p* = 0.023) (Figure 3B). Cows fed RPM also had greater mRNA abundance of *KEAP1* (1.15 vs. 0.92; *p* = 0.017) and *CUL3* (1.36 vs. 1.03; *p* = 0.009) relative to control cows. Feeding RPM [40] led to greater *NFE2L2* abundance in mammary gland tissue during early lactation. The protein encoded by *NFE2L2*, a regulator of transcription of antioxidant genes [41], has been associated with the abundance of antioxidant gene networks, including *KEAP1* and *CUL3* [21,42]; hence, an increase in *NFE2L2* suggested greater activation of antioxidant systems and is likely one of the mechanisms behind the changes in *KEAP1* and *CUL3* mRNA abundance. Under quiescent/homeostatic conditions, *NFE2L2* is constantly degraded via the *KEAP1*-mediated ubiquitin-proteasome pathway. However, under stress conditions, *NFE2L2* translocates into the nucleus and binds to antioxidant response elements of the target cytoprotective genes. Studies using *NFE2L2*-deficient mice on a C57BL/6 background demonstrated that *NFE2L2* is important for antioxidant enzymes in skeletal muscles [43]. Thus, we speculate that a similar role for this transcription regulator exists in the skeletal muscle of dairy cows.

### 3.7. CDP-Choline Pathway

Despite the lack of diet × time interaction for genes involved in the CDP-Choline pathway, cows fed RPM had greater expression of *CHKB* (1.70 vs. 1.12; *p* = 0.006), *PCYT1A* (1.46 vs. 0.97; *p* = 0.001) and *PCYT1B* (1.85 vs. 1.25; *p* = 0.001) compared with controls (Table 2). Rumen-protected Met supplementation during the transition period is expected to increase not only AA balance for milk synthesis but also promote the synthesis of important methylated compounds, including phosphatidylcholine (PC), which can be achieved through the CDP-choline pathway [22]. Choline kinases (*CHKA* and *CHKB*) catalyze the first committed step in phosphatidylcholine synthesis from choline [44]. The choline taken up into cells is readily trapped as phosphocholine [44]. Thus, the greater *CHKA* in cows fed RPM suggested an enrichment of the phosphocholine pool during the periparturient period. A similar upregulation of *CHKA* was detected in periparturient cows supplemented with rumen-protected choline starting at ~4 weeks prepartum [22]. It is noteworthy, however, that mRNA abundance of *CHKA*, *CHKB* and *PCYT1B* did not change, and *PCYT1A* was lower in dairy cow primary liver cells cultured with Met at 40 μM compared with controls (Met-free medium) [45]. In a study with 3T3-L1 and NIH-3T3 cells, decreased abundance of *PCYT1A* [the rate-limiting enzyme for phosphatidylcholine (PC) synthesis] led to an inhibition of PC synthesis [46]. Thus, the observed increase in *PCYT1A* and *PCYT1B* in cows supplemented with RPM suggested that it led to greater availability of CDP-choline to synthesize PC. The tendency for a greater abundance of *CEPT1*, which catalyzes the final step of PC synthesis via the Kennedy pathway, when RPM was fed supports this idea. Overall, these results suggested that the biosynthetic flux from choline to PC is responsive to RPM. It is likely that the greater DMI in cows fed RPM [6] contributed to increased PC synthesis and potentially insulin sensitivity, as demonstrated in humans [47]. It is well-known that dairy cows are insulin insensitive, especially around parturition, when the mammary gland’s needs for nutrients, including AA and glucose, are the greatest. Thus, the positive effects of RPM on muscle PC may bring additional benefits to the cow by enhancing insulin sensitivity and helping prevent excessive muscle breakdown.

### 3.8. Arginine Metabolism

There was no diet × time effect for genes associated with arginine metabolism (Table 2). However, the abundance of *ARG1* and *NOS2* was greater (*p* = 0.004 and *p* = 0.005) in cows fed RPM (1.96 and 1.31) compared with controls (0.53 and 0.38). Furthermore, RPM also led to greater mRNA abundance of *SRM*, *SMS* (both *p* < 0.05) and *NOS3* (*p* < 0.001). Primary bovine mammary epithelial cells supplemented with Met at 70 µg/mL medium had lower mRNA abundance of *NOS2*, but no effect was detected on mRNA abundance of *ODC1*, *SRM*, *AMD1*, and *ARG1* compared with controls containing 60 µg of Met/mL in the medium [23]. Arginine plays an important role in cell signaling in skeletal muscle tissue. For example, in a cell culture study with mouse C2C12 myoblasts, L-Arg was associated with the regulation of muscle development via the mTOR (Thr 2446)/p70S6K signaling pathway in a NO-dependent manner [48]. Thus, regulation of intracellular protein turnover via Arg can favor the maintenance of muscle mass in livestock species [49]. At present, few studies have been conducted to assess quantitative aspects of Arg synthesis and catabolism in the whole body or tissues of ruminants. It is noteworthy, however, that supplementation of Arg via the jugular vein during early lactation alleviated inflammation and metabolic stress in dairy cows [50]. The fact that the abundance of both *SLC25A29* and *SCL38A9* was greater in skeletal muscle of cows fed RPM suggested that Arg content might have been enriched.

In summary, feeding RPM during the transition period positively affected the abundance of nearly all target genes analyzed. Although this AA led to greater DMI around parturition [6] that could have altered mRNA abundance as in mice [51], the enhanced post-ruminal supply of Met might directly stimulate molecular alterations in skeletal muscle through a number of mechanisms, including the rate of translation.

## 4. Conclusions

Feeding ethyl-cellulose RPM during the periparturient period in dairy cows increases mRNA abundance of skeletal muscle genes associated with the transport of several nutrients and biological processes that generate energy, replenish tissue protein, and coordinate antioxidant responses. Thus, besides positive effects at the level of performance, RPM can help skeletal muscle maintain homeostasis around parturition partly through changes in mRNA abundance.

## Figures and Tables

**Table 1:** Ingredient and nutrient composition of close-up (from −28 d to parturition) and early lactation (from 1 to 60 DIM) diets.

Item	Close-Up Diet	Fresh Diet
Feed ingredient (% DM)		
Alfalfa haylage	6.55	7.81
Corn silage	26.6	31.0
Wheat straw	26.5	3.25
Corn grain (ground, dry)	12.6	22.2
Cottonseed	-	2.17
Molasses (beet sugar)	4.03	5.50
Soybean hulls	3.46	4.25
Soybean meal (48 % CP)	7.83	10.1
Expeller soybean meal ^1^	5.80	5.16
Protein supplement ^2^	0.78	1.81
Urea	0.59	0.39
Soychlor ^3^	1.23	
Saturated fat supplement ^4^	-	2.25
Limestone	-	1.41
Salt	-	0.02
Dicalcium phosphate	0.52	1.17
Magnesium oxide	-	0.08
Magnesium sulfate	2.08	0.02
Sodium bicarbonate	-	0.84
Mineral vitamin mix ^5^	0.17	0.17
Vitamin A (30,000 kIU/kg)	0.03	0.02
Vitamin D (5000 kIU/kg)	0.03	-
Vitamin E (44,000 kIU/kg)	0.60	-
Biotin ^6^	0.70	0.42
Ethyl-cellulose RPM ^7^	0.09	0.10
Chemical composition		
CP, %	15.7	17.7
NDF, %	40.7	29.3
ADF, %	27.4	19.6
EE, %	2.33	5.11
Mcal NE_L_/kg	1.47	1.67

^1^ SoyPlus, West Central Soy (Ralston, IA). ^2^ ProVAAl AADvantage, Perdue AgriBusiness (Salisbury, MD, USA). ^3^ West Central Soy. ^4^ Energy Booster 100, Milk Specialties Global (Eden Prairie, MN, USA). ^5^ Contained a minimum of 5% Mg, 10% S, 7.5% K, 2.0% Fe, 3.0% Zn, 3.0% Mn, 5000 mg of Cu/kg, 250 mg of I/kg, 40 mg of Co/kg, 150 mg of Se/kg, 2200 kIU of vitamin A/kg, 660 kIU of vitamin D3/kg, and 7700 IU of vitamin E/kg. ^6^ ADM Animal Nutrition (Quincy, IL, USA). ^7^ Ethyl-cellulose rumen-protected methionine, Evonik Nutrition and Care GmbH (Hanau-Wolfgang, Germany) added only in the RPM group.

**Table 2:** mRNA expression of genes in skeletal muscle of dairy cows (*n* = 10/diet) fed a basal control diet or the basal diet plus ethyl-cellulose rumen-protected methionine (0.9 g/kg DMI at prepartum and 1.0 g/kg DMI at postpartum) from −21 to 21 day of calving. Statistical *p*-values for the main effect of time are reported in the figures in the Supplemental File (Figures S1–S7).

Gene	Description	Diet		SEM	*p*-Value	
		Control	RPM		Diet	Diet × Time
Amino acid transport						
*SLC1A5*	Neutral amino acid transporter	1.24	1.44	0.25	0.346	0.339
*SLC3A2*	Heavy-chain amino acid transporter	1.18	1.52	0.13	0.064	0.601
*SLC7A5*	Branched-chain and aromatic amino acid transporter	0.91	0.99	0.45	0.863	0.330
*SLC7A8*	Branched-chain and aromatic amino acid transporter	0.86	1.60	0.45	0.047	0.035
*SLC38A1*	Neutral amino acid transporter	0.96	1.41	0.29	0.132	0.293
*SLC38A2*	Neutral amino acid transporter	1.06	1.30	0.14	0.098	0.074
*SLC38A6*	Sodium-dependent amino acid transporter	1.26	1.52	0.29	0.118	0.067
*SLC38A7*	Glutamate and serine transporter, Gln, His, Ser, Ala, Asn	1.15	1.32	0.22	0.339	0.268
*SLC43A2*	L-amino acid transporter-3 (Leu, Phe, Val, Met)	1.02	1.61	0.23	0.003	0.006
*SLC25A29*	Mitochondrial transporter of basic AA (Arg and Lys)	1.95	3.87	0.84	0.016	0.681
*SCL38A9*	Gln, Leu, and Arg transporter	0.95	1.37	0.22	0.017	0.447
Carnitine transport and β-oxidation						
*SLC22A5*	Sodium-dependent high-affinity carnitine transporter	0.85	1.04	0.08	0.030	0.022
*CPT1A*	Carnitine palmitoyltransferase 1A	1.02	1.38	0.29	0.068	0.382
*ACADVL*	Acyl-CoA dehydrogenase very long chain	0.98	1.36	0.17	0.013	0.191
Vitamin transport						
*SLC5A6*	Multivitamin transporter	1.14	1.86	0.38	0.006	0.267
*SLC19A2*	Thiamin transporter	1.20	1.70	0.19	0.013	0.076
*SLC44A1*	Choline transporter	1.05	1.25	0.16	0.082	0.174
mTOR/insulin signaling						
*AKT1*	AKT serine/threonine kinase 1	0.90	1.23	0.16	0.006	0.438
*mTOR*	Mechanistic target of rapamycin kinase	1.02	1.26	0.15	0.033	0.623
*IRS1*	Insulin receptor substrate 1	0.88	1.27	0.12	<0.001	0.002
Antioxidant response						
*NFE2L2*	NFE2-like bZIP transcription factor 2	0.96	1.43	0.17	0.003	0.010
*KEAP1*	Kelch-like ECH-associated protein 1	0.92	1.15	0.12	0.017	0.552
*CUL3*	Cullin 3	1.03	1.36	0.15	0.009	0.396
CDP-Choline pathway						
*CHKA*	Choline kinase alpha	1.06	1.91	0.44	0.066	0.356
*CHKB*	Choline kinase beta	1.12	1.70	0.19	0.006	0.391
*PCYT1A*	Phosphate cytidylyltransferase 1A, choline	0.97	1.46	0.14	0.001	0.792
*PCYT1B*	Phosphate cytidylyltransferase 1B, choline	1.25	1.85	0.15	0.001	0.119
*CEPT1*	Choline/ethanolamine phosphotransferase 1	1.15	1.86	0.35	0.054	0.130
Arginine metabolism						
*ODC1*	Ornithine decarboxylase 1	0.97	1.10	0.17	0.440	0.628
*SRM*	Spermidine synthase	1.12	1.34	0.10	0.031	0.556
*AMD1*	Adenosylmethionine decarboxylase 1	1.12	1.33	0.16	0.198	0.234
*ARG1*	Arginase 1	0.53	1.96	0.43	0.004	0.370
*SMS*	Spermine synthase	0.80	0.98	0.08	0.020	0.444
*NOS2*	Nitric oxide synthase 2	0.38	1.31	0.29	0.005	0.323
*NOS3*	Nitric oxide synthase 3	0.71	1.11	0.11	<0.001	0.257

## Data Availability

The data presented in this study are available upon reasonable request from the corresponding author.

## Supplementary Materials

The following are available at https://www.mdpi.com/article/10.3390/ani12131641/s1. Table S1: GenBank accession number, hybridization position, sequence, and amplicon size of designed primers for Bos taurus used to analyze gene expression. Table S2: Sequence results of 16 PCR products from primers of genes used in the experiment. Table S3: Best hit using BLAST Genomes (bovine genome) Search and BLASTN of NCBI. Table S4: RT-qPCR performance for the 38 genes measured in skeletal muscle. Figure S1: Expression of genes associated with amino acid transport in skeletal muscle of dairy cows fed a basal control diet or the basal diet plus ethyl-cellulose rumen-protected methionine from −21 to 21 day of calving. Figure S2: Expression of genes associated with fatty acid β-oxidation in skeletal muscle of dairy cows fed a basal control diet or the basal diet plus ethyl-cellulose rumen-protected methionine from −21 to 21 day of calving. Figure S3: Expression of genes associated with vitamin transport in skeletal muscle of dairy cows fed a basal control diet or the basal diet plus ethyl-cellulose rumen-protected methionine from −21 to 21 day of calving. Figure S4: Expression of genes associated with mTOR/insulin signaling in skeletal muscle of dairy cows fed a basal control diet or the basal diet plus ethyl-cellulose rumen-protected methionine from −21 to 21 day of calving. Figure S5: Expression of genes associated with antioxidant response in skeletal muscle of dairy cows fed a basal control diet or the basal diet plus ethyl-cellulose rumen-protected methionine from −21 to 21 day of calving. Figure S6: Expression of genes associated with CDP-Choline pathway in skeletal muscle of dairy cows fed control and ethyl-cellulose rumen-protected methionine diets from −21 to 21 day of calving. Figure S7: Expression of genes associated with arginine metabolism in skeletal muscle of dairy cows fed a basal control diet or the basal diet plus ethyl-cellulose rumen-protected methionine from −21 to 21 day of calving.

## References

[1] NRC (2001). National Research Council: Nutrient Requirements of Dairy Cattle.

[2] Komaragiri M.V., Erdman R.A. (1997). Factors affecting body tissue mobilization in early lactation dairy cows. 1. Effect of dietary protein on mobilization of body fat and protein. J. Dairy Sci..

[3] Komaragiri M.V., Casper D.P., Erdman R.A. (1998). Factors affecting body tissue mobilization in early lactation dairy cows. 2. Effect of dietary fat on mobilization of body fat and protein. J. Dairy Sci..

[4] Grummer R.R. (1995). Impact of changes in organic nutrient metabolism on feeding the transition dairy cow. J. Anim. Sci..

[5] Bell A.W., Burhans W.S., Overton T.R. (2000). Protein nutrition in late pregnancy, maternal protein reserves and lactation performance in dairy cows. Proc. Nutr. Soc..

[6] Batistel F., Arroyo J.M., Bellingeri A., Wang L., Saremi B., Parys C., Trevisi E., Cardoso F.C., Loor J.J. (2017). Ethyl-cellulose rumen-protected methionine enhances performance during the periparturient period and early lactation in Holstein dairy cows. J. Dairy Sci..

[7] Fehlberg L.K., Guadagnin A.R., Thomas B.L., Sugimoto Y., Shinzato I., Cardoso F.C. (2020). Feeding rumen-protected lysine prepartum increases energy-corrected milk and milk component yields in Holstein cows during early lactation. J. Dairy Sci..

[8] Tebbe A.W., Weiss W.P. (2021). Concurrent and carryover effects of feeding blends of protein and amino acids in high-protein diets with different concentrations of forage fiber to fresh cows. 1. Production and blood metabolites. J. Dairy Sci..

[9] Sadri H., Ghaffari M.H., Schuh K., Dusel G., Koch C., Prehn C., Adamski J., Sauerwein H. (2020). Metabolome profiling in skeletal muscle to characterize metabolic alterations in over-conditioned cows during the periparturient period. J. Dairy Sci..

[10] Tebbe A.W., Hanson J., Weiss W.P. (2021). Effects of metabolizable protein concentration, amino acid profile, and fiber source on the messenger RNA expression of skeletal muscle in peripartum dairy cows. J. Dairy Sci..

[11] Toledo M.Z., Stangaferro M.L., Gennari R.S., Barletta R.V., Perez M.M., Wijma R., Sitko E.M., Granados G., Masello M., Van Amburgh M.E. (2021). Effects of feeding rumen-protected methionine pre- and postpartum in multiparous Holstein cows: Lactation performance and plasma amino acid concentrations. J. Dairy Sci..

[12] Osorio J.S., Ji P., Drackley J.K., Luchini D., Loor J.J. (2014). Smartamine M and MetaSmart supplementation during the peripartal period alter hepatic expression of gene networks in 1-carbon metabolism, inflammation, oxidative stress, and the growth hormone-insulin-like growth factor 1 axis pathways. J. Dairy Sci..

[13] Osorio J.S., Ji P., Drackley J.K., Luchini D., Loor J.J. (2013). Supplemental Smartamine M or MetaSmart during the transition period benefits postpartal cow performance and blood neutrophil function. J. Dairy Sci..

[14] Zhou Z., Vailati-Riboni M., Trevisi E., Drackley J.K., Luchini D.N., Loor J.J. (2016). Better postpartal performance in dairy cows supplemented with rumen-protected methionine compared with choline during the peripartal period. J. Dairy Sci..

[15] Schwab C.G., Wallace R.J., Chesson A. (1995). Protected proteins and amino acids for ruminants. Biotechnology in Animal Feeds and Animal Feeding.

[16] Overton T.R., LaCount D.W., Cicela T.M., Clark J.H. (1996). Evaluation of a ruminally protected methionine product for lactating dairy cows. J. Dairy Sci..

[17] Coleman D.N., Vailati-Riboni M., Elolimy A.A., Cardoso F.C., Rodriguez-Zas S.L., Miura M., Pan Y.-X., Loor J.J. (2019). Hepatic betaine-homocysteine methyltransferase and methionine synthase activity and intermediates of the methionine cycle are altered by choline supply during negative energy balance in Holstein cows. J. Dairy Sci..

[18] Batistel F., Alharthi A.S., Wang L., Parys C., Pan Y.-X., Cardoso F.C., Loor J.J. (2017). Placentome nutrient transporters and mammalian target of rapamycin signaling proteins are altered by the methionine supply during late gestation in dairy cows and are associated with newborn birth weight. J. Nutr..

[19] Hu L., Chen Y., Cortes I.M., Coleman D.N., Dai H., Liang Y., Parys C., Fernandez C., Wang M., Loor J.J. (2020). Supply of methionine and arginine alters phosphorylation of mechanistic target of rapamycin (mTOR), circadian clock proteins, and α-s1-casein abundance in bovine mammary epithelial cells. Food Funct..

[20] Naeem A., Drackley J.K., Stamey J., Loor J.J. (2012). Role of metabolic and cellular proliferation genes in ruminal development in response to enhanced plane of nutrition in neonatal Holstein calves. J. Dairy Sci..

[21] Han L.Q., Zhou Z., Ma Y., Batistel F., Osorio J.S., Loor J.J. (2018). Phosphorylation of nuclear factor erythroid 2-like 2 (NFE2L2) in mammary tissue of Holstein cows during the periparturient period is associated with mRNA abundance of antioxidant gene networks. J. Dairy Sci..

[22] Zhou Z., Garrow T.A., Dong X., Luchini D.N., Loor J.J. (2017). Hepatic activity and transcription of betaine-homocysteine methyltransferase, methionine synthase, and cystathionine synthase in periparturient dairy cows are altered to different extents by supply of methionine and choline. J. Nutr..

[23] Dai H., Coleman D.N., Hu L., Martinez-Cortés I., Wang M., Parys C., Shen X., Loor J.J. (2020). Methionine and arginine supplementation alter inflammatory and oxidative stress responses during lipopolysaccharide challenge in bovine mammary epithelial cells in vitro. J. Dairy Sci..

[24] Ding L.Y., Chen L.M., Wang M.Z., Zhang J., Loor J.J., Zhou G., Zhang X., Wang H.R. (2018). Inhibition of arginase via jugular infusion of N(ω)-hydroxy-nor-l-arginine inhibits casein synthesis in lactating dairy cows. J. Dairy Sci..

[25] Bionaz M., Loor J.J. (2007). Identification of reference genes for quantitative real-time PCR in the bovine mammary gland during the lactation cycle. Physiol. Genom..

[26] Kuhla B., Nürnberg G., Albrecht D., Görs S., Hammon H.M., Metges C.C. (2011). Involvement of skeletal muscle protein, glycogen, and fat metabolism in the adaptation on early lactation of dairy cows. J. Proteome Res..

[27] Sun F., Cao Y., Cai C., Li S., Yu C., Yao J. (2016). Regulation of Nutritional Metabolism in Transition Dairy Cows: Energy Homeostasis and Health in Response to Post-Ruminal Choline and Methionine. PLoS ONE.

[28] Zhou Z., Riboni M.V., Bulgari O., Trevisi E., Garrow T.A., Drackley J.K., Cardoso P., Luchini D.N., Loor J.J. (2016). Physiological and molecular mechanisms associated with performance, immunometabolic status, and liver function in transition dairy cows fed rumen-protected methionine or choline. J. Anim. Sci..

[29] Zou K., Ouyang Q., Li H., Zheng J. (2017). A global characterization of the translational and transcriptional programs induced by methionine restriction through ribosome profiling and RNA-seq. BMC Genom..

[30] Lager S., Powell T.L. (2012). Regulation of nutrient transport across the placenta. J. Pregnancy.

[31] Stephens F.B., Constantin-Teodosiu D., Greenhaff P.L. (2007). New insights concerning the role of carnitine in the regulation of fuel metabolism in skeletal muscle. J. Physiol..

[32] Kato Y., Sugiura M., Sugiura T., Wakayama T., Kubo Y., Kobayashi D., Sai Y., Tamai I., Iseki S., Tsuji A. (2006). Organic cation/carnitine transporter OCTN2 (Slc22a5) is responsible for carnitine transport across apical membranes of small intestinal epithelial cells in mouse. Mol. Pharmacol..

[33] Ringseis R., Keller J., Eder K. (2018). Regulation of carnitine status in ruminants and efficacy of carnitine supplementation on performance and health aspects of ruminant livestock: A review. Arch. Anim. Nutr..

[34] Schäff C., Börner S., Hacke S., Kautzsch U., Sauerwein H., Spachmann S.K., Schweigel-Röntgen M., Hammon H.M., Kuhla B. (2013). Increased muscle fatty acid oxidation in dairy cows with intensive body fat mobilization during early lactation. J. Dairy Sci..

[35] Javed K., Fairweather S.J. (2019). Amino acid transporters in the regulation of insulin secretion and signalling. Biochem. Soc. Trans..

[36] Hay N., Sonenberg N. (2004). Upstream and downstream of mTOR. Genes Dev..

[37] Wullschleger S., Loewith R., Hall M.N. (2006). TOR signaling in growth and metabolism. Cell.

[38] González I.M., Martin P.M., Burdsal C., Sloan J.L., Mager S., Harris T., Sutherland A.E. (2012). Leucine and arginine regulate trophoblast motility through mTOR-dependent and independent pathways in the preimplantation mouse embryo. Dev. Biol..

[39] Wang P., Drackley J.K., Stamey-Lanier J.A., Keisler D., Loor J.J. (2014). Effects of level of nutrient intake and age on mammalian target of rapamycin, insulin, and insulin-like growth factor-1 gene network expression in skeletal muscle of young Holstein calves. J. Dairy Sci..

[40] Han L., Batistel F., Ma Y., Alharthi A.S.M., Parys C., Loor J.J. (2018). Methionine supply alters mammary gland antioxidant gene networks via phosphorylation of nuclear factor erythroid 2-like 2 (NFE2L2) protein in dairy cows during the periparturient period. J. Dairy Sci..

[41] Suzuki T., Yamamoto M. (2015). Molecular basis of the Keap1-Nrf2 system. Free Radic. Biol. Med..

[42] Elolimy A.A., Liang Y., Lopes M.G., Loor J.J. (2021). Antioxidant networks and the microbiome as components of efficiency in dairy cattle. Livest. Sci..

[43] Kitaoka Y. (2021). The role of Nrf2 in skeletal muscle on exercise capacity. Antioxidants.

[44] Fagone P., Jackowski S. (2013). Phosphatidylcholine and the CDP-choline cycle. Biochim. Biophys. Acta.

[45] Zhou Y.F., Zhou Z., Batistel F., Martinez-Cortés I., Pate R.T., Luchini D.L., Loor J.J. (2018). Methionine and choline supply alter transmethylation, transsulfuration, and cytidine 5′-diphosphocholine pathways to different extents in isolated primary liver cells from dairy cows. J. Dairy Sci..

[46] Okazaki Y., Nakamura K., Takeda S., Yoshizawa I., Yoshida F., Ohshima N., Izumi T., Klein J.D., Kumrungsee T., Sands J.M. (2019). GDE5 inhibition accumulates intracellular glycerophosphocholine and suppresses adipogenesis at a mitotic clonal expansion stage. Am. J. Physiol. Cell Physiol..

[47] Newsom S.A., Brozinick J.T., Kiseljak-Vassiliades K., Strauss A.N., Bacon S.D., Kerege A.A., Bui H.H., Sanders P., Siddall P., Wei T. (2016). Skeletal muscle phosphatidylcholine and phosphatidylethanolamine are related to insulin sensitivity and respond to acute exercise in humans. J. Appl. Physiol..

[48] Wang R., Jiao H., Zhao J., Wang X., Lin H. (2018). L-Arginine enhances protein synthesis by phosphorylating mTOR (Thr 2446) in a nitric oxide-dependent manner in C2C12 cells. Oxid. Med. Cell. Longev..

[49] Wu G., Bazer F.W., Satterfield M.C., Gilbreath K.R., Posey E.A., Sun Y. (2022). L-Arginine nutrition and metabolism in ruminants. Adv. Exp. Med. Biol..

[50] Ding L.Y., Wang Y.F., Shen Y.Z., Zhou G., Wu T.Y., Zhang X., Wang M.Z., Loor J.J., Zhang J. (2020). Effects of intravenous arginine infusion on inflammation and metabolic indices of dairy cows in early lactation. Animal.

[51] Saladin R., De Vos P., Guerre-Millot M., Leturque A., Girard J., Staels B., Auwerx J. (1995). Transient increase in obese gene expression after food intake or insulin administration. Nature.

