# Supplementary material for: Alterations in Skeletal Muscle mRNA Abundance in Response to Ethyl-Cellulose Rumen-Protected Methionine during the Periparturient Period in Dairy Cows
Source: Animals (Basel). 2022 Jun 26;12(13):1641. doi: 10.3390/ani12131641 (PMC9264958; doi:10.3390/ani12131641)
Supplement: Supplementary file 1 [file animals-12-01641-s001.zip › animals-1741421-supplementary.pdf]

Table S1. GenBank accession number, hybridization position, sequence, and amplicon size of designed primers for *Bos taurus* used to analyze gene expression

Gene	Accession #	Primers F/R	Forward primers (5'-3')	Reverse primers (5'-3')	Amplicon size (bp)	Source
SLC1A5	NM_174601.2	F.23/R.151	GGCTAGCAGCTGTTTACTCCT	AGTCTGGGGGCTAGAAGACG	129	In this article
SLC3A2	NM_001024488.2	F.131/R.305	GAGCATTCCCTTGCTTGCAC	GCTCATGGTGCCTGAGTCG	175	In this article
SLC7A5	NM_174613.2	F.1205/R.1385	CCGTACCCTCACTGGTGTTC	AGATGAACCTTGATGGGCCG	181	In this article
SLC7A8	NM_001192889.2	F.18/R.160	GCACCGGAACAACACTGAAA	CAGAGCCGATGATGTTCCCTAC	143	In this article
SLC38A1	XM_010827702.2	F.2257/R.2424	GGAAGGGCGGATACCACTTT	TGACACCCCTGTTATCTCAGC	168	In this article
SLC38A2	NM_001082424.1	F.461/R.608	TGAAAAGCCATTATGCCGATGT	CCCACAATCGCATTGCTCAG	148	In this article
SLC38A7	NM_001100355.1	F.1379/R.1492	CTTTGTCTTCCCAGGGCTGT	CCCAGTGTGACCAAGAGGAC	114	In this article
SLC43A2	NM_001075546.1	F.1200/R.1342	TACACCTCCATCTTCGGGGT	TTCTTCTCACCTGGGTTGGC	143	In this article
SLC25A29	NM_001077871.1	F.1744/R.1855	ACCCCTACTCCTTCCACTCC	ATCTCCCCTTCCACTGACCT	131	In this article
SLC38A9	NM_001102163	-	GGGCATAAAACAGGCTGGGT	CTTGACCACTGCCCAAAGGA	183	19
SLC22A5	NM_001046502.2	F.817/R.918	GCAGCATTTGTCCTGGGAAC	CAGCAGCATGTAGCCAAACG	102	In this article
CPT1A	FJ415874.1	F.141/R.240	TCGCGATGGACTTGCTGTATA	CGGTCCAGTTTGCGTCTGTA	100	20
ACADVL	NM_001078076.1	F.707/R.768	CCAGCCCCTGTGGAAAATACTA	GCCCCCGTTACTGATCCAA	124	20
SLC5A6	NM_001046219.2	F.1366/R.1533	CCCTGACCAGTTCGTCTTGT	GGGAACCAGGGTCGAATCAA	168	In this article
SLC19A2	NM_001206010.1	F.930/R.1040	AGTCGTGAACTACGCACAGG	ACTGCAACAGCACCCAGTAA	111	In this article
SLC44A1	XM_015464658.1	F.294/R.415	CAGGTGCAGCAGCAAGACTA	TACATACTTCCGATGGGTGTGG	122	In this article
AKT1	NM_173986.2	F.115/R.248	CTGCACAAGCGAGGTGAGTA	GAGAAGTTGTTGAGGGGCGA	134	In this article
mTOR	NM_001144096.3	F.4145/R.4278	GGTTAACACCAAGCAGGTTCAT	GTATGTCGCACTGGACACCA	134	In this article
IRS1	XM_003581871.4	F.3904/R.4090	CTCAAGAGTGCCCACCTCAA	AGGTCTTCATTCTGCTGTGAT	187	In this article
NFE2L2	NM_001011678	-	TACCTGGGAGTAGTTGGCA	ACAACAGTGTGGAGAGGTATGAGC	108	21
KEAP1	NM_001101142	-	ACAACAGTGTGGAGAGGTATGAGC	AGAGCAGACGGTTGAGGACAG	110	21
CUL3	XM_010802427.2	F.1279/R.1370	ACTGGGGTATCTTTAGGTGGTG	ATGTCTTGGTGCTGGTGGGA	111	In this article
CHKA	XM_002699402	F.534/R.663	GCACAGGTTCCTCAGTTA	GCCATCCAGCAGTAAGAT	130	22
CHKB	XM_010805827	F.1239/R.1401	CCAAGAGGAGCAGAGGAA	GTAGAACTGGAACCGAGACT	163	22

Gene	Accession #	Primers F/R	Forward primers (5'-3')	Reverse primers (5'-3')	Amplicon size (bp)	Source
PCYT1A	XM_005201384	F.899/R.1062	GATGAGGTGGTGAGGAAT	CGAGTGATGATGTCTGATG	197	22
PCYT1B	NM_001193051	F.413/R.597	ACTGTCGCTATGTGGATGAA	GCCTTCTGTTCTCTGTGTTG	185	22
CEPT1	NM_001193130	F.1329/R.1528	GGATAGCCCTGGTCTTCTCTT	ATTCGCCTCAATATGTTCAGATTCTT	200	22
ODC1	NM_174130.2	F.833/R.852	CGCTGTGTCTTTGACATGGG	TGTCCAGTGCTGGGTTGATT	136	23
SRM	NM_001303623.1	F.688/R.707	AAGGAGGACGGCATTCTCTG	AAGTGCCGCATCTCCTTGAT	77	23
AMD1	NM_173990.2	F.354/R.425	GAGGTTTGGTTCTCCAGGCA	GGATCTTGGGATGGTGCGAA	72	23
ARG1	NM_001046154.1	F.1371/R.1447	TCAGGAGACAGAGCTACCCA	AGCAGTTTCTAGGCATTACAGC	77	23
SMS	NM_001035471.1	F.129/R.259	CAGCACCCTCGACTTCATGCT	TCGCTAAATAGCCATGGTCCTG	131	23
NOS2	NM_001076799.1	F.1004/R.1124	GGTACGAATGGTTCCGGGAG	CCCATGTACCACCCGTTGAA	121	23
NOS3	XM_024990490.1	F.1681/R.1865	GCATGGATGAGTATGACGTGGTGT	GAAGCGGATCTTGTAACTCTTGTGC	185	24

Table S2. Sequence results of 16 PCR products from primers of genes used in the experiment.

Gene	PCR product sequence	bp
SLC1A5	ATAGGCGGCCCCCTTGGGCCCCGGGGCTTGAATCTCCCCTCCTTGGACCCAAAAACCTGGCCCCCTCTCTTCCCGGAGCTCCGTCTTTCTAGCCCCCAGACA<br>CAAAC	107
SLC3A2	AAAAAACGCGGGCAGGGTCCTACTCCATGGGACCGCGAGCCTCCTGAACCCTCCGCTGGCGTCATCTCGGTTCCAAGCCAGCCGCCCAGCGAGCCTTCTGG<br>GCCTGGTCCTCAGGTGCCCAGCGCGGGGGGCGACTCAGGCACCATGAGCAA	152
SLC7A5	CTTCTCCGTCATCAACTTTCTTNAGCTTCTTCAACTGGCTCTGCGTGGCCCTGGCCATTNGCTGGCATGCTCTGGCTTCGCTACCAGAAACCGGAGCTGGAGC<br>GGCNNNNAAGGTTCA	118
SLC7A8	TCAAAGGGAACGNNNCGCAGGAAGGCTGATTCTGGGAGGGGGCGGAGTGGCCCTGAAGAAAGAGATTGGATTGGTCAGTGGTTGTGCTATCATCGTAGGG<br>AACATCATCGGCTCTCGA	118
SLC38A1	CTCCTTTTGCCAGAAACTTATCAAGTGTCTACTGTGTGCCAGGCACTGTATCTGTGTGCATAGAAAAACCCCTGAAGCTGATACTCCAATATACATATACAG<br>TGATCATCTCTGCTTGCTGAGATAACAGGGGTGTCAAAC	141
SLC38A2	TTAANAACGAACTTTTTACTTGAATCGAATTTGGGGAAGAAGAAGTATGAAACTGACTTTCATCCAGGTACTACTTCCTTTGGAATGTCAGTATTTAATCTG<br>AGCAATGCGATTGTGGGAATTAGATTTCAAGTCTAACTTGCCCAAAGCTGGTTTTCTCCATTTAATTATTGTTATATGTTTGGTTATCCTTTTCCCTCTATGTA<br>GCATCCAGGTACTACTTCCTTTGGAATGTCAGTATTTAATCTGAGCAATGCGATTGTGGGACTA	270
SLC38A7	TCACCAATAATNNCTGAAATGGAGGAANTNAAGCCAGCCAGCNGGTGGGCGATGGTCAGTTACGGAGTCCTCCTTGGTCACTCTCAGGTNGATATAAGGTG<br>ATGATTACTGTTCTGAAAAAAGCATTTGTATCAAAANATTGATGGTTTATGTATCGGCAAGGAACTACCAAAAAAAAATTGAGGGGAATGTCTN	195
SLC43A2	AACCCNCGCCGCTCCGCCCCGTCATTGGCTACATCATGGGATTGGAGGCTGAAGGAGTGTGAGGATGCCTCCGAGGAGCCCGAGGAGAAAGACGCCAACC<br>CAGGTGAG	108
SLC25A29	ATAGNTCTGGGGCCCTAAGTAAGCCATGGAACCTTTCTGGAAGTTCTACATTAGCTTAGGAGCTGGGGTGGGGGGGCAGGCACAGGTCAGTGGAAGGGGA<br>GTATCAATT	109
SLC5A6	ACCTGGAGATCCCTGGANNGAGCCTGCCGGGGCTGCCTGGGGCTCCTTCGTCGCCTGCCCTCTTCAGTGGCTCCCTCAGCACCATATCCTCTGCTTTTAATTC<br>ACTGGCAACTGTTACAATGGAAGACTTGATTCGACCCTTGGTTCCCACNA	153
SLC19A2	CCCGGGAAGGGAAGCCTTCCCGCCAATGCTGCTTATCAATAAATGGGCGGTGTGGAGGCCGTTTCAACCTTACTGGGGTGCTGTTTTGAGAGGGTGAAACA<br>ACTCCACCCACATTAAAAACACCGAAAGGATCACAGAGTNCACGGCACCGTGATCACTAACAAAATGAAACNNNTGAATGTGATACTGGTAGAAGGTCTC<br>AACCANNNCAATAACGATGACTAGTATTAACACACTCCCGCGCGTGCACTACAGAGGATGTTGCAGAGTAGGATTGA	278
SLC44A1	ACAGNAGAGCTAATGGGAAATATCTGTGGGCAGAAAATGCAGAGTTGGAAGCAATACCAAACAGTGGCATGGACCACACCCATCGGAAGTATGTATAGAN<br>NT	102
AKT1	CNNNAAAGTGGCGGCCGGCTTTACTTCCCTCCTGAAGAACGNATGGGCACGTTTCATTGGGCTACAAGGAGCGGCCGCAGGACCTGGAGCAGCGTGAGTC<br>GCCCCTCAACNAACTTTCTCAANAGNNACCCTTTTNNAANACATAGACGNCAACAGACCNGCAGATACACTATTGATTGGGACCATCGGAGTTGTCGCGCT<br>CCGCGGTCACTTGGTCGGGTTCCTTGAGTGCTACTCATATCACAATCGGACTCCTGATCTTATTTATCATCAGTTTGGCCGGAAACGCGCAGTGCTTACAAA<br>ATACTGCAAATCAGCACTTCATACCAGGAGCCAAACGAGAGGTAGGCATCGTTTGAGNNNAGAGCATGGTTTATNNCTACTCGGCCGCTGCAAGANGGAA<br>TTCCATGTACTTCACGGGTAAGTAATGGCTNNGGCTTTGAGAAGAACNNTAGCATTTTTTTTTTTTTAAGAGGNNTAGTNNNCGCGGAGCCAGGTTTTTGAC<br>GATAGGGTACAAGGCCCCCCCCCACTTCGNNNGCTGGGAAGCACTTAGGCCTTCTCCTAGGAAGAGCCAAGCCTGCCTGGGCCGNTACATACGCCACCACA<br>GCCATCGAATAGTGCTCAAAGTGTTCGGGAATGAGAACCNNNNACCGAGTACCAATTTGCCAGTAGGTANGTATAATCCGCTGA	690
MTOR	TCGCTAGNNNNNNAGNNTTATTAAGTCCTATTAACCAAAATACGCTGCAGCGCTCCNNNTNCGTGAGGTCCATGGTGTCCAGTGCGACA	89
IRS1	TCTCCCCCCTCCGGGCCCTCATCAGCCTCTGGCCAGTAGTCAGAGCGGCCCCACCAGCCGCTCCAGCGAGGATCTAAGCGCCTATGCCAGCATCACTTTCCA<br>GAAGCAGCCAGAGGACCTCCAGTAGCTCAACTGGACATCACAGCAGAAATGAAGACCTAAAA	164
CUL3	TCAGGGGTTGGGNTGGTNTAAGACAGGTAAACCTGGGTGGCACTCAGTCAGCCGACACCAAAGTGCAACATCCCACCAGCACCAAGACANNNCCGCATCA<br>CTCGTGTTACCCCGTAATAAC	121

Table S3. The table reports the best hit using BLAST Genomes (bovine genome) Search and BLASTN of NCBI

Gene	Primers	NCBI					
		Genbank	Best hit	Score	E value	Per. Ident, %	Accession #
SLC1A5	F.23	SLC1A5	Bos taurus solute carrier family 1 member 5	137	1e-28	92.00	NM_174601.2
SLC3A2	F.131	SLC3A2	Bos taurus solute carrier family 3 member 2	254	2e-63	98.61	NM_001024488.2
SLC7A5	F.1205	SLC7A5	Bos taurus solute carrier family 7 member 5	180	3e-41	97.17	NM_174613.2
SLC7A8	F.18	SLC7A8	Bos taurus solute carrier family 7 member 8	172	4e-39	98.00	NM_001192889.2
SLC38A1	F.2257	SLC38A1	Bos taurus solute carrier family 38 member 1	231	9e-57	99.22	XM_002687321.5
SLC38A2	F.461	SLC38A2	Bos taurus solute carrier family 38 member 2	316	1e-48	100.00	NM_001082424.1
SLC38A7	F.1379	SLC38A7	Bos taurus solute carrier family 38 member 7	102	1e-17	92.65	NM_001100355.1
SLC43A2	F.1200	SLC43A2	Bos taurus solute carrier family 43 member 2	165	7e-37	97.92	NM_001075546.1
SLC25A29	F.1744	SLC25A29	Bos taurus solute carrier family 25 member 29	156	4e-34	95.10	NM_001077871.1
SLC5A6	F.1366	SLC5A6	Bos taurus solute carrier family 5 member 6	211	1e-50	96.88	NM_001046219.2
SLC19A2	F.930	SLC19A2	Bos taurus solute carrier family 19 member 2	97.1	8e-16	89.87	NM_001206010.3
SLC44A1	F.294	SLC44A1	Bos taurus solute carrier family 44 member 1	147	2e-31	96.67	XM_024996305.1
AKT1	F.115	AKT1	Bos taurus AKT serine/threonine kinase 1	152	5e-32	92.04	NM_173986.2
MTOR	F.4145	RICTOR	Bos taurus RPTOR independent companion of MTOR complex 2	119	4e-23	94.44	NM_001144096.3
IRS1	F.3904	IRS1	Bos taurus insulin receptor substrate 1	272	7e-69	96.95	XM_003581871.4
CUL3	F.1279	CUL3	Bos taurus isolate L1 Dominette 01449 registration number	84.2	1e-14	97.96	NC_037329.1

Table S4. RT-qPCR performance among the 38 genes measured in skeletal muscle of dairy cows (*n*=10/diet)

Function and gene symbol	Median Ct(1)	Median ΔCt(2)	Slope(3)	R^{2}(4)	Efficiency(5)	relative mRNA abundance(6)	1/EΔCt(7)	%(8)
Amino acid transport								
SLC1A5	26.62	5.91	-3.160	0.979	2.072	0.013	0.005	0.54
SLC3A2	26.80	6.00	-3.174	0.986	2.065	0.013	0.005	0.52
SLC7A5	28.97	8.18	-3.041	0.957	2.133	0.002	0.001	0.08
SLC7A8	25.04	4.43	-3.265	0.948	2.024	0.044	0.018	1.76
SLC38A1	27.06	6.38	-3.409	0.994	1.965	0.013	0.005	0.54
SLC38A2	22.09	1.31	-3.434	0.992	1.955	0.415	0.166	16.6
SLC38A6	27.26	6.49	-3.129	0.992	2.087	0.008	0.003	0.34
SLC38A7	26.59	5.78	-3.197	0.994	2.055	0.016	0.006	0.62
SLC43A2	22.79	1.88	-3.423	0.991	1.960	0.281	0.113	11.3
SLC25A29	29.15	8.32	-3.372	0.998	1.979	0.003	0.001	0.14
SCL38A9	26.50	5.70	-3.183	0.997	2.061	0.016	0.006	0.65
Carnitine transport and β-oxidation								
SLC22A5	27.41	6.54	-3.20	0.991	2.052	0.009	0.004	0.36
CPT1A	26.93	6.01	-3.004	0.992	2.152	0.010	0.004	0.40
ACADVL	22.99	2.26	-3.187	0.997	2.060	0.196	0.078	7.83
Vitamin transport								
SLC5A6	28.11	7.39	-3.357	0.988	1.986	0.006	0.003	0.25
SLC19A2	28.02	7.32	-3.193	0.985	2.057	0.005	0.002	0.20
SLC44A1	25.50	4.76	-3.026	0.997	2.140	0.027	0.011	1.07
mTOR/insulin signaling								
AKT1	24.94	4.15	-3.563	0.995	1.909	0.068	0.027	2.73
mTOR	25.56	4.84	-3.328	0.992	1.997	0.035	0.014	1.41
IRS1	23.94	3.19	-3.564	0.997	1.908	0.127	0.051	5.09
Antioxidant response								
NFE2L2	24.17	3.19	-3.245	0.998	2.033	0.104	0.041	4.15
KEAP1	23.46	2.71	-3.549	0.990	1.913	0.172	0.069	6.87
CUL3	23.17	2.45	-3.220	0.999	2.044	0.174	0.070	6.96
CDP-Choline pathway								
CHKA	26.86	5.99	-3.01	0.935	2.148	0.010	0.004	0.41
CHKB	23.80	2.93	-3.22	0.996	2.043	0.123	0.049	4.93
PCYT1A	25.36	4.49	-3.42	0.989	1.962	0.048	0.019	1.94

Function and gene symbol	Median Ct[(1)]	Median ΔCt[(2)]	Slope[(3)]	$R^{2(4)}$	Efficiency[(5)]	relative mRNA abundance[(6)]	1/EΔCt[(7)]	%[(8)]
PCYT1B	25.10	4.24	-3.05	0.971	2.127	0.041	0.016	1.63
CEPT1	24.93	4.06	-3.07	0.958	2.115	0.048	0.019	1.91
Arginine metabolism								
ODC1	22.98	2.11	-3.45	0.980	1.951	0.244	0.097	9.74
SRM	25.85	4.98	-3.26	0.987	2.025	0.030	0.012	1.19
AMD1	26.02	5.15	-3.22	0.996	2.044	0.025	0.010	1.01
ARG1	31.07	10.20	-3.01	0.996	2.152	0.000	0.000	0.02
SMS	23.50	2.63	-3.35	0.993	1.987	0.164	0.066	6.57
NOS2	30.81	9.95	-3.16	0.995	2.071	0.001	0.000	0.03
NOS3	27.70	6.83	-3.15	0.984	2.078	0.007	0.003	0.27
Reference/housekeeping								
GAPDH	16.31		-3.45	0.997				
RPS9	21.68		-3.42	0.994				
UXT	25.44		-3.48	0.992				

SLC1A5: Neutral amino acid transporter; SLC3A2: Heavy-chain amino acid transporter; SLC7A5: Branched-chain and aromatic amino acid transporter; SLC7A8: Branched-chain and aromatic amino acid transporter; SLC38A1: Neutral amino acid transporter; SLC38A2: Neutral amino acid transporter; SLC38A6: Sodium-dependent amino acid transporter; SLC38A7: Glutamate and serine transporter, Gln, His, Ser, Ala, Asn; SLC43A2: L-amino acid transporter-3 (Leu, Phe, Val, Met); SLC25A29: Mitochondrial transporter of basic AA (Arg and Lys); SCL38A9: Gln, Leu and Arg transporter; SLC22A5: Sodium-dependent high affinity carnitine transporter; CPT1A: Carnitine palmitoyltransferase 1A; ACADVL: Acyl-CoA dehydrogenase very long chain; SLC5A6: Multivitamin transporter; SLC19A2: Thiamin transporter; SLC44A1: Choline transporter; AKT1: AKT serine/threonine kinase 1; mTOR: Mechanistic target of rapamycin kinase; IRS1: Insulin receptor substrate 1; NFE2L2: NFE2 like bZIP transcription factor 2; KEAP1: Kelch like ECH associated protein 1; CUL3: Cullin 3; CHKA: Choline kinase alpha; CHKB: Choline kinase beta; PCYT1A: Phosphate cytidylyltransferase 1A, choline; PCYT1B: Phosphate cytidylyltransferase 1B, choline; CEPT1: Choline/ethanolamine phosphotransferase 1; ODC1: Ornithine decarboxylase 1; SRM: Spermidine synthase; AMD1: Adenosylmethionine decarboxylase 1; ARG1: Arginase 1; SMS: Spermine synthase; NOS2: Nitric oxide synthase 2; NOS3: Nitric oxide synthase 3; GAPDH: Glyceraldehyde 3-phosphate dehydrogenase; RPS9: Ribosomal protein S9; UXT: Ubiquitously expressed prefoldin like chaperone.

[1] The median is calculated considering all time points and all cows.

[2] The median of ΔCt is calculated as [Ct gene – geometrical mean of Ct internal controls] for each time point and each cow.

[3] Slope of the standard curve.

[4] R^2 stands for the coefficient of determination of the standard curve.

[5] Efficiency is calculated as $[10^{(-1 / Slope)}]$.

[6] relative mRNA abundance = 1/ Efficiency $^{\text{Median } \Delta Ct}$

[7] 1/EΔCt = relative mRNA abundance/∑relative mRNA abundance

[8] % = (1/EΔCt)*100

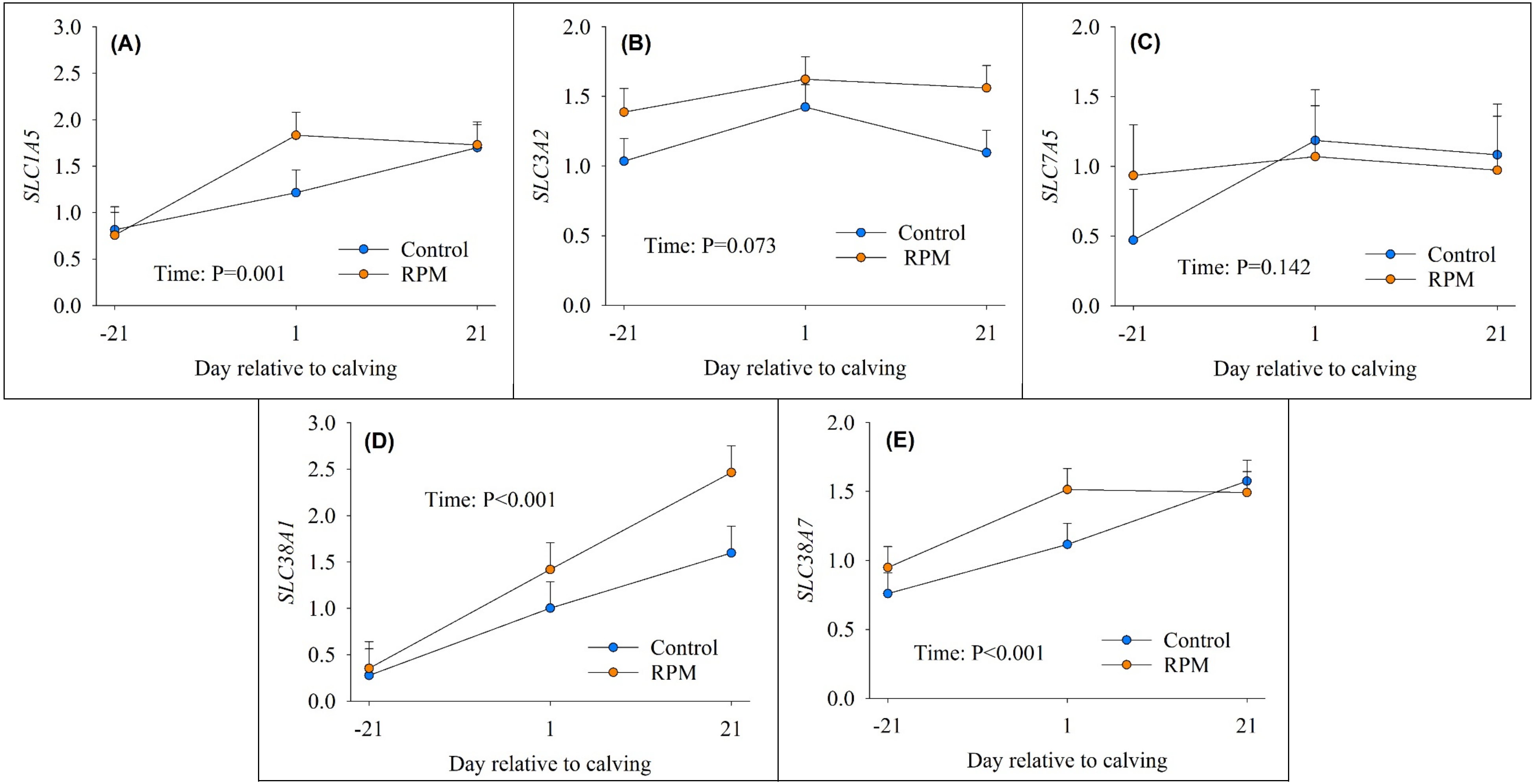
(A)
SLC1A5
Time: P=0.001
Control
RPM
Day relative to calving
(B)
SLC3A2
Time: P=0.073
Control
RPM
Day relative to calving
(C)
SLC7A5
Time: P=0.142
Control
RPM
Day relative to calving
(D)
SLC38A1
Time: P<0.001
Control
RPM
Day relative to calving
(E)
SLC38A7
Time: P<0.001
Control
RPM
Day relative to calving

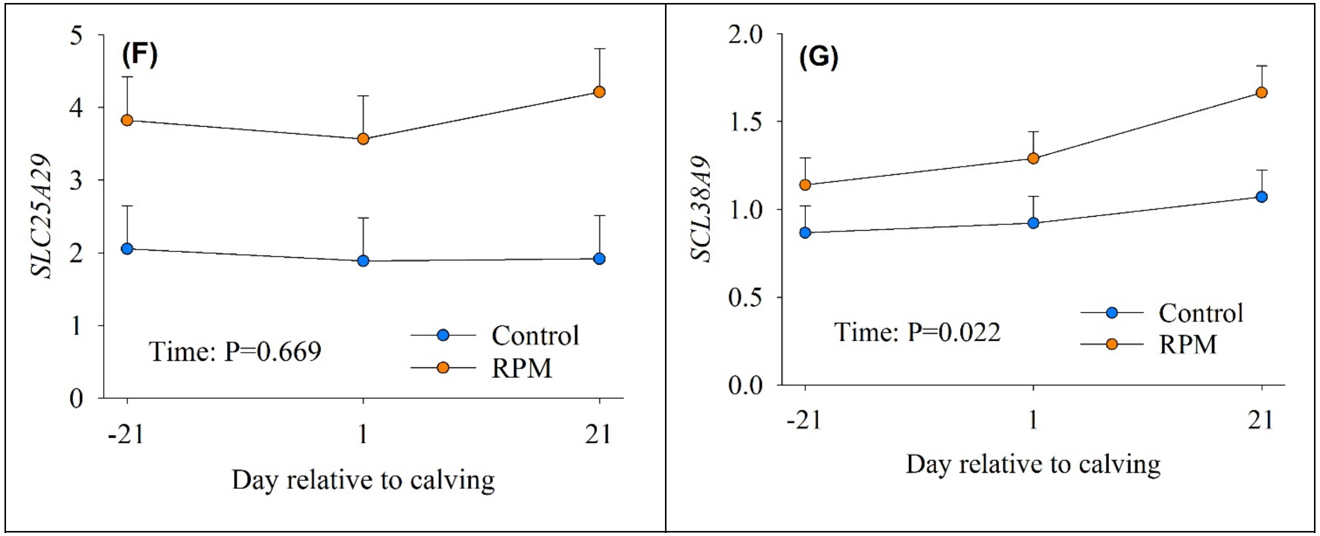

Figure S1. Expression of genes associated with amino acid transport in skeletal muscle of dairy cows (*n*=10/diet) fed a basal control diet or the basal diet plus ethyl-cellulose rumen-protected methionine from -21 to 21 day of calving. Error bars represent SEM.

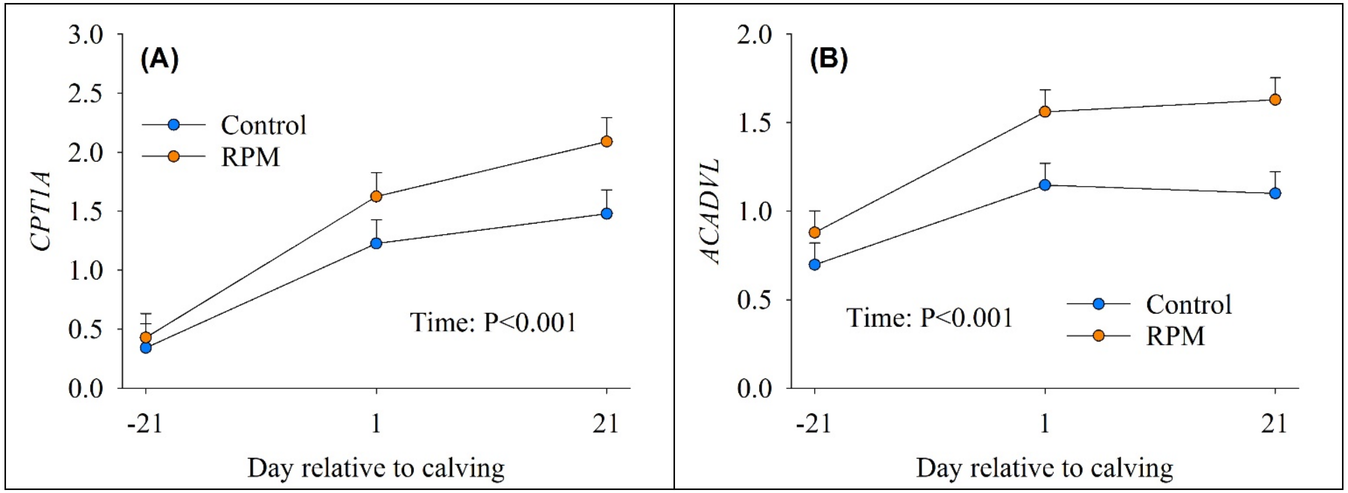

Figure S2. Expression of genes associated with fatty acid β-oxidation in skeletal muscle of dairy cows (*n*=10/diet) fed a basal control diet or the basal diet plus ethyl-cellulose rumen-protected methionine from -21 to 21 day of calving. Error bars represent SEM.

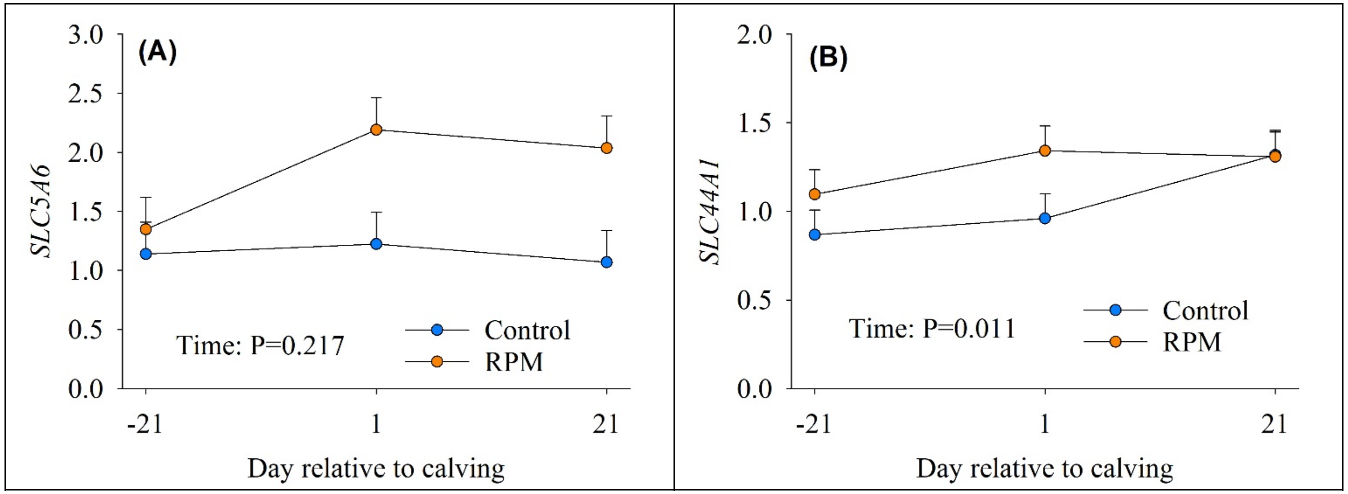

Figure S3. Expression of genes associated with vitamin transport in skeletal muscle of dairy cows (*n*=10/diet) fed a basal control diet or the basal diet plus ethyl-cellulose rumen-protected methionine from -21 to 21 day of calving. Error bars represent SEM.

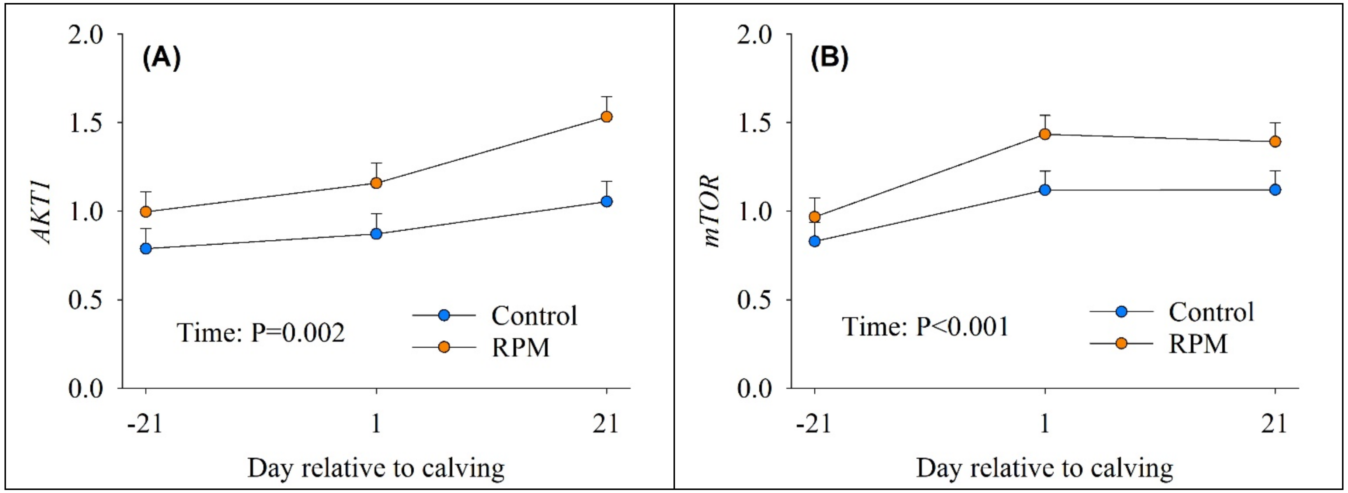

Figure S4. Expression of genes associated with mTOR/insulin signaling in skeletal muscle of dairy cows (*n*=10/diet) fed a basal control diet or the basal diet plus ethyl-cellulose rumen-protected methionine from -21 to 21 day of calving. Error bars represent SEM.

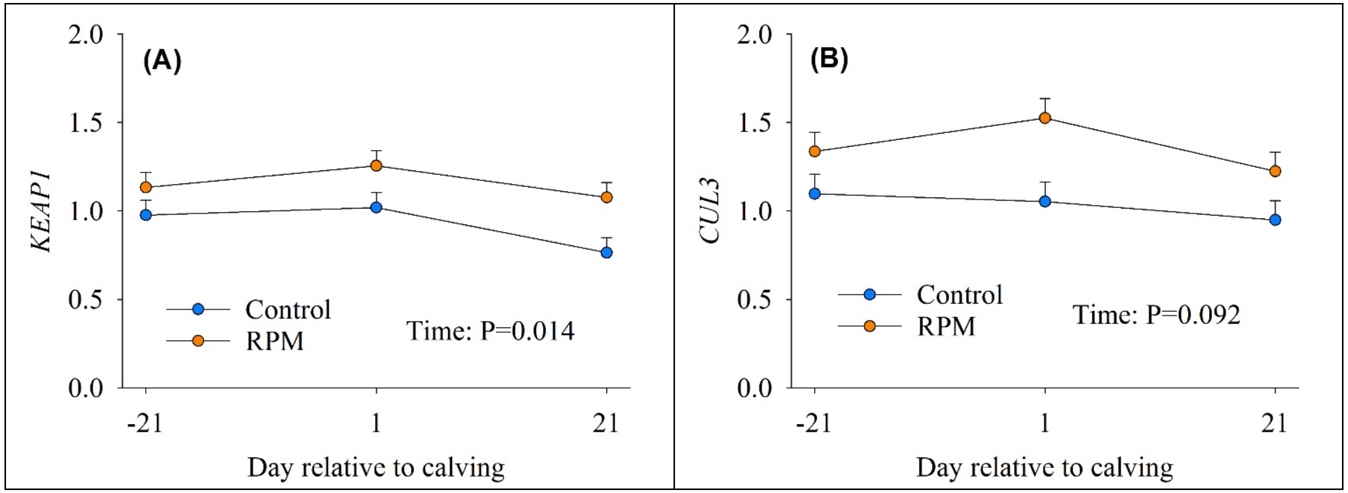

Figure S5. Expression of genes associated with antioxidant response in skeletal muscle of dairy cows (*n*=10/diet) fed a basal control diet or the basal diet plus ethyl-cellulose rumen-protected methionine from -21 to 21 day of calving. Error bars represent SEM.

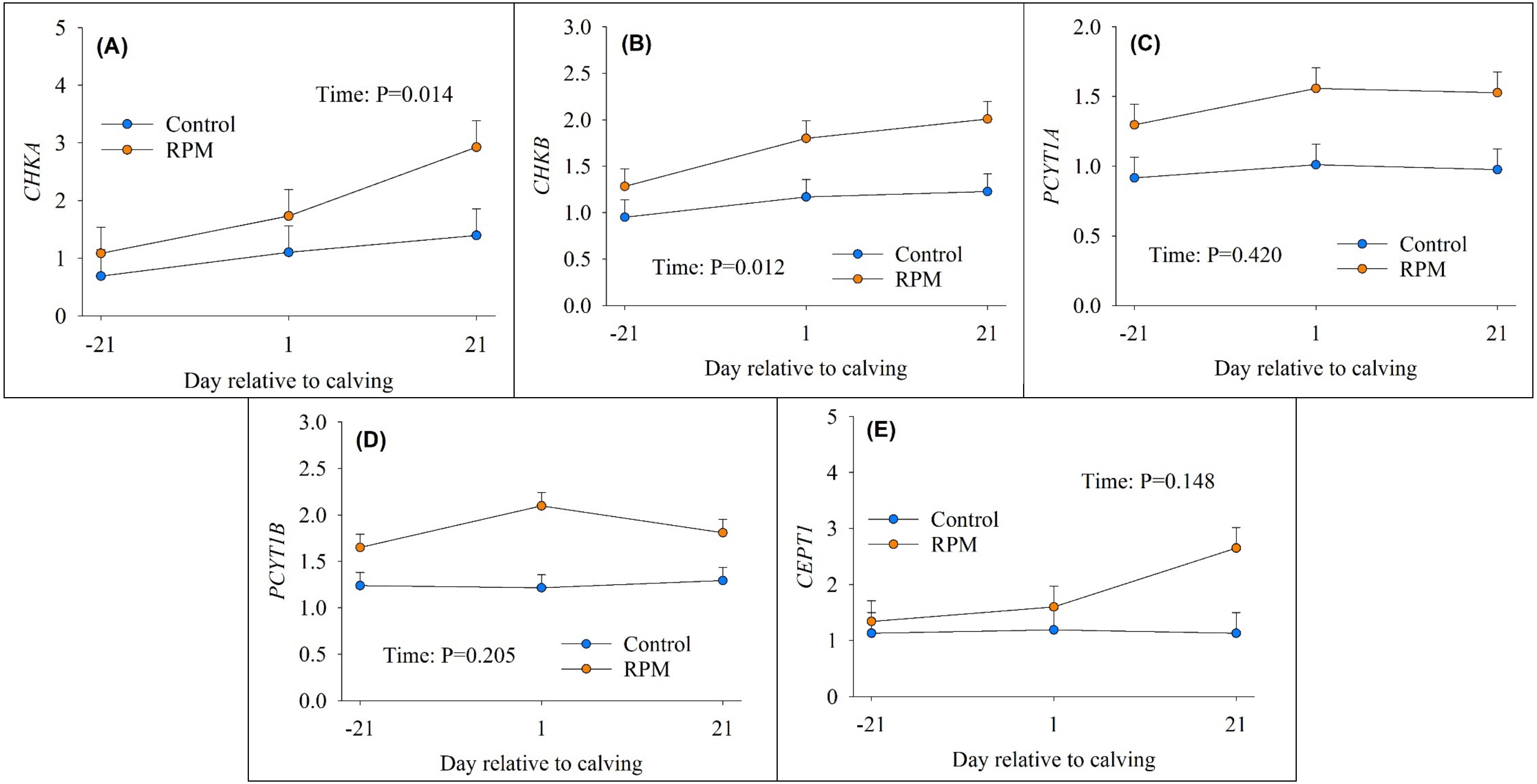

Figure S6. Expression of genes associated with CDP-Choline pathway in skeletal muscle of dairy cows (*n*=10/diet) fed control and ethyl-cellulose rumen-protected methionine diets from -21 to 21 day of calving. Error bars represent SEM.

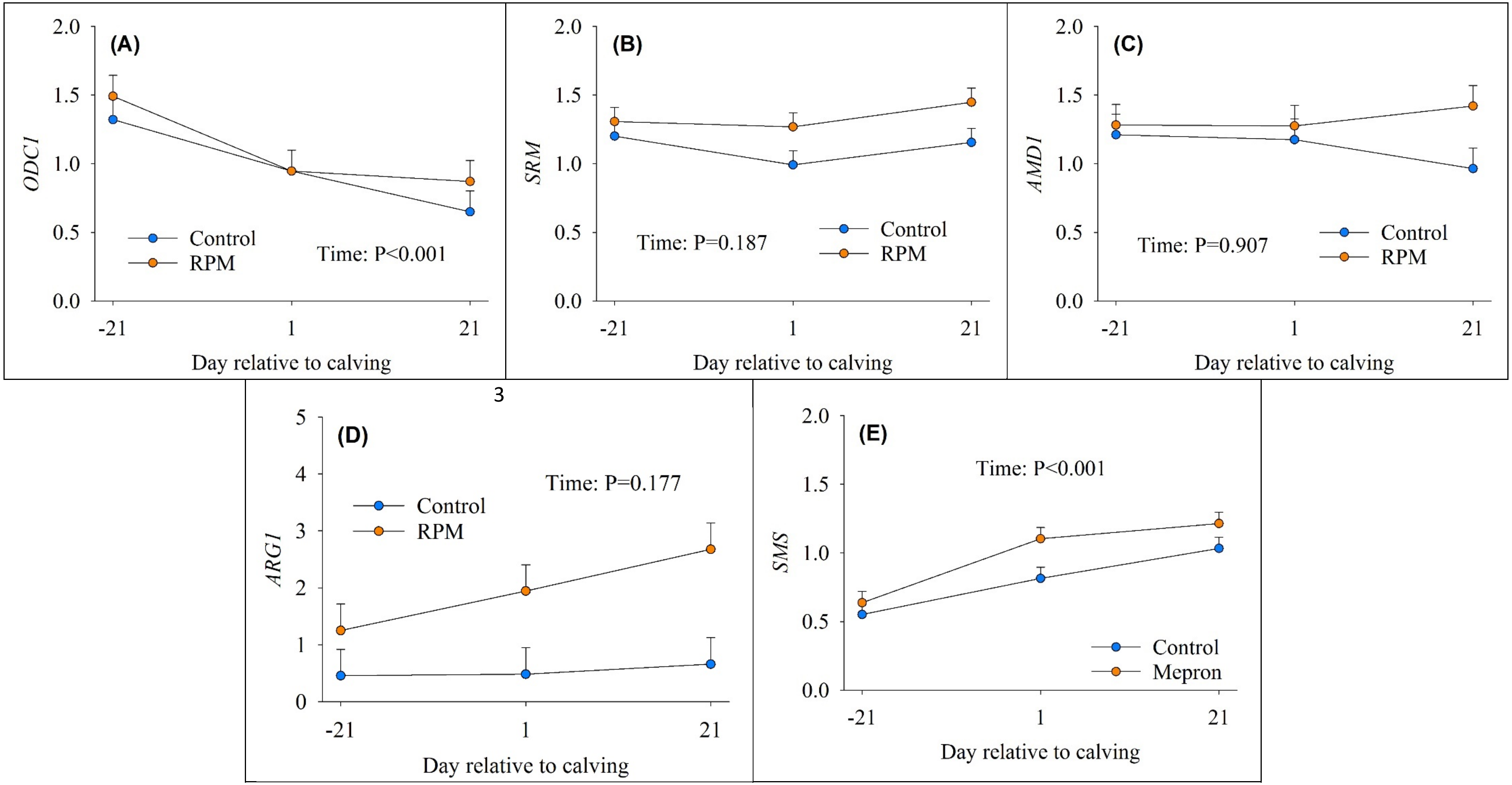
(A)
ODC1
2.0
1.5
1.0
0.5
0.0
Control
RPM
Time: P<0.001
-21
1
21
Day relative to calving
(B)
SRM
Time: P=0.187
Control
RPM
Day relative to calving
(C)
AMD1
Time: P=0.907
Control
RPM
Day relative to calving
3
(D)
ARG1
5
4
3
2
1
0
Time: P=0.177
Control
RPM
Day relative to calving
(E)
SMS
Time: P<0.001
Control
Mepron
Day relative to calving

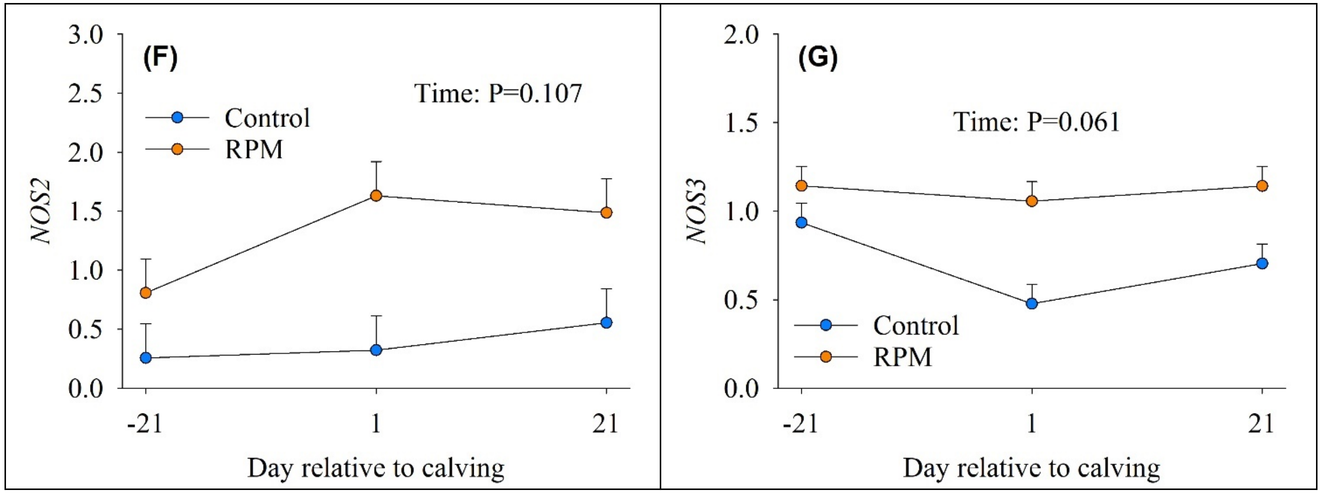

Figure S7. Expression of genes associated with arginine metabolism in skeletal muscle of dairy cows (*n*=10/diet) fed a basal control diet or the basal diet plus ethyl-cellulose rumen-protected methionine from -21 to 21 day of calving. Error bars represent SEM.
